# The moss-specific transcription factor PpERF24 positively modulates immunity against fungal pathogens in *Physcomitrium patens*

**DOI:** 10.3389/fpls.2022.908682

**Published:** 2022-09-15

**Authors:** Guillermo Reboledo, Astrid Agorio, Lucía Vignale, Alfonso Alvarez, Inés Ponce De León

**Affiliations:** ^1^Departamento de Biología Molecular, Instituto de Investigaciones Biológicas Clemente Estable, Montevideo, Uruguay; ^2^Laboratorio de Fisiología Vegetal, Facultad de Ciencias, Centro de Investigaciones Nucleares, Universidad de la República, Montevideo, Uruguay

**Keywords:** transcription factor, AP2/ERF, *Physcomitrium patens*, pathogens, defense

## Abstract

APETALA2/ethylene response factors (AP2/ERFs) transcription factors (TFs) have greatly expanded in land plants compared to algae. In angiosperms, AP2/ERFs play important regulatory functions in plant defenses against pathogens and abiotic stress by controlling the expression of target genes. In the moss *Physcomitrium patens*, a high number of members of the ERF family are induced during pathogen infection, suggesting that they are important regulators in bryophyte immunity. In the current study, we analyzed a *P. patens* pathogen-inducible ERF family member designated as PpERF24. Orthologs of PpERF24 were only found in other mosses, while they were absent in the bryophytes *Marchantia polymorpha* and *Anthoceros agrestis*, the vascular plant *Selaginella moellendorffii*, and angiosperms. We show that PpERF24 belongs to a moss-specific clade with distinctive amino acids features in the AP2 domain that binds to the DNA. Interestingly, all *P. patens* members of the PpERF24 subclade are induced by fungal pathogens. The function of PpERF24 during plant immunity was assessed by an overexpression approach and transcriptomic analysis. Overexpressing lines showed increased defenses to infection by the fungal pathogens *Botrytis cinerea* and *Colletotrichum gloeosporioides* evidenced by reduced cellular damage and fungal biomass compared to wild-type plants. Transcriptomic and RT-qPCR analysis revealed that PpERF24 positively regulates the expression levels of defense genes involved in transcriptional regulation, phenylpropanoid and jasmonate pathways, oxidative burst and pathogenesis-related (PR) genes. These findings give novel insights into potential mechanism by which PpERF24 increases plant defenses against several pathogens by regulating important players in plant immunity.

## Introduction

Plant transcription factors (TFs) play crucial roles in regulating the expression of target genes that function in developmental processes and responses against environmental stresses. APETALA2/ethylene response factors (AP2/ERFs) are a large plant-specific TF superfamily that participates in growth, development, and defense responses to abiotic and biotic stress in angiosperms (Xu et al., 2011). The number of AP2/ERF TFs has greatly expanded in land plants compared to algae, suggesting that they may have contributed to terrestrialization (Bowman et al., 2017). While algae have between six and 27 AP2/ERF genes, most land plants have more than 100 members according to the PlantTFDB v5.0 database (Jin et al., 2017). Members of the family are defined by the AP2 domain, which comprises one or two AP2 DNA-binding domains with 60–70 conserved amino acid residues that consist of three β-sheet strands and an α-helix motif (Allen et al., 1998). The AP2 domain has two important regions, namely, YRG and RAYD elements. The YRG element includes 20 amino acids that play a critical role in establishing direct contact with the DNA (Okamuro et al., 1997). The RAYD element contains approximately 40 amino acids in the α-helix region that plays an important role in protein–protein interaction, as well as a putative alternative role in DNA binding (Jofuku et al., 1994; Okamuro et al., 1997). Based on the number of AP2 domains and sequence similarities, AP2/ERFs are classified into three major families, including APETALA2 (AP2), ethylene-responsive element binding protein (ERF), and related to ABI3/VP (RAV) (Licausi et al., 2013). A fourth group belonging to the AP2/ERF superfamily is named Soloist, and although it contains a single AP2 domain, sequence and gene structure strongly diverge from those of the AP2/ERF TFs (Sakuma et al., 2002). The ERF family is further divided into two major subfamilies, the ERF and the dehydration-responsive element-binding (DREB) subfamily, which comprises 12 groups in *Arabidopsis thaliana* (Sakuma et al., 2002; Nakano et al., 2006). AP2 TFs have two tandem AP2 domains, whereas ERF and DREB family members contain a single AP2 domain (Nakano et al., 2006). Usually, AP2 members are key factors that regulate development and organ formation (Aukerman and Sakai, 2003), while members of the ERF and DREB subfamily are more related to responses to environmental stress (Lata and Prasad, 2011). DREBs bind to the dehydration-responsive element (DRE) in stress-responsive genes and are mainly involved in plant tolerance against different types of abiotic stresses, including drought, freezing, salt, and heat (Lata and Prasad, 2011). In contrast, ERFs are involved in defense responses to pathogen attack by binding to a *cis*-acting ethylene response element (ERE: GCC box) (Solano et al., 1998; Berrocal-Lobo et al., 2002). The amino acids valine at position 14 and glutamic acid at position 19 (V14 and E19) in the AP2 domain are important for binding to DRE elements, while alanine at position 14 (A14) and aspartic acid at position 19 (D19) are important for binding to ERE elements (Sakuma et al., 2002). More recently, it was shown that several ERFs also bind to DRE elements and play regulatory roles in integrating ethylene, abscisic acid (ABA), jasmonate, and redox signaling in plant responses to abiotic stresses (Müller and Munné-Bosch, 2015).

Several studies have revealed that overexpression of ERF TFs in angiosperms increases resistance against different pathogens and tolerance to drought, cold, and salt (Xu et al., 2011; Wang et al., 2022). For example, overexpression of some ERFs increased resistance to *Stemphylium lycopersici* in tomato (Yang H. et al., 2020), *Fusarium solani* in potato (Charfeddine et al., 2019), *Botryosphaeria dothidea* in apple (Wang et al., 2020), *Rhizoctonia cerealis* in wheat (Chen et al., 2008), and *Magnaporthe oryzae* and *Xanthomonas oryzae* pv. *oryzae* in rice (Hong et al., 2022). Increased resistance was associated with enhanced expression of pathogenesis-related (PR) proteins (Chen et al., 2008; Charfeddine et al., 2019). Moreover, *DREB DEAR1* expression increased in response to pathogen infection, and its overexpression in *A. thaliana* renders plants with improved resistance to the bacterial pathogen *Pseudomonas syringae* (Tsutsui et al., 2009).

Mosses, together with liverworts and hornworts, are bryophytes that represent the three major lineages of non-vascular plants. During plant evolution, bryophytes have adapted to terrestrial environments with variations in temperature, UV-B radiation, desiccation stress, and exposure to fungi and bacteria (Rensing et al., 2008; Ponce de León and Montesano, 2017). Adaptation mechanisms to these changing environments resulted in the acquisition of novel genetic information. Comparative studies of bryophytes and vascular plants allow the identification of gene losses and duplications events within gene families, including TFs, and their functional conservation or diversification among different plant lineages. Additionally, evolutionary conserved biological roles of genes during stress and their presence in the most recent common ancestor of land plants can be assessed. In the alga *Chlamydomonas reinhardtii*, an *AP2/ERF* gene participates in the early response to cold stress (Li L. et al., 2020), suggesting that the involvement of AP2/ERF members in defense responses to stress occurs throughout the green lineages. In contrast to angiosperms, studies on the functional role of moss TFs in stress mechanisms have not been addressed until the present. The moss *Physcomitrium patens*, formerly *Physcomitrella patens*, is considered a model plant to study abiotic and biotic stresses in mosses (Frank et al., 2005; Ponce de León and Montesano, 2017). According to the PlantTFDB v5.0 database, *P. patens* has 161 AP2/ERF genes, divided into 16 AP2, 143 ERF (ERF + DREB), and two RAV. This number is comparable with other plants, such as *A. thaliana*, *Solanum lycopersicum*, and *Oryza sativa*, which have 146, 167, and 157 members, respectively. Transcriptomic analyses revealed that *P. patens* differentially expresses many members of the AP2/ERF superfamily in response to biotic interactions and abiotic stresses (Reboledo et al., 2022). *Botrytis cinerea* and *Colletotrichum gloeosporioides* inoculation led to differential expression of a high number of AP2/ERFs compared to control tissues, including 90 ERF/DREB (69 induced and 21 repressed), 7 AP2 (6 induced and 1 repressed), and one RAV that was only induced with *B. cinerea* (Reboledo et al., 2022). These findings suggest that ERF and DREB TFs are important regulators in *P. patens* defense responses against biotic stress.

In a previous study, we identified a member of the *P. patens* ERF family, which was induced during treatments with elicitors of the phytopathogenic bacteria *Pectobacterium carotovorum* subsp. *carotovorum* (Alvarez et al., 2016). Transcriptomic analysis of *P. patens* tissues during biotic and abiotic stresses revealed that this gene is highly induced by fungal pathogens, while it is repressed by ABA and drought (Reboledo et al., 2022), highlighting a possible involvement in plant responses to pathogen infection. Here, we assessed the role of this TF, named as PpERF24, during plant immunity by an overexpression approach and transcriptomic analysis. We show that PpERF24 positively modulates plant defenses against fungal pathogens by regulating expression levels of genes related to defense.

## Materials and methods

### Plant material and growth conditions

*P. patens* Gransden wild type and PpERF24 overexpressing lines were grown and maintained on solid BCDAT medium (Ashton and Cove, 1977) with cellophane disks under standard long-day conditions (22°C, 16-h light/8-h dark regime under 60–80 μmol m^2^ s^–1^ white light). Three-week-old colonies were used for all experiments.

### Pathogen inoculation and culture filtrate treatments

The necrotrophic bacteria *P. carotovorum* strain SCC1 (Rantakari et al., 2001) was propagated in Luria–Bertani medium at 28°C. Cell-free culture filtrates (CFs) were prepared according to Ponce de León et al. (2007). The CF containing *P.c. carotovorum* elicitors was applied on *P. patens* colonies by spray. *B. cinerea* was cultivated on Potato Dextrose Agar (PDA) at 22°C, and 2 × 10^5^ spores/mL suspension was used to inoculate *P. patens* colonies by spray (Ponce de León et al., 2007). *C. gloeosporioides* was grown on PDA at 22°C, and 2 × 10^5^ conidia/mL suspension was used to inoculate *P. patens* colonies by spray (Reboledo et al., 2015). Water-treated plants were used as control. *Pythium irregulare* was grown on PDA at 26°C, and *P. patens* colonies were inoculated with 0.5 cm diameter agar plugs containing *P. irregulare* mycelium, as described by Oliver et al. (2009). As control, PDA plugs were placed on top of each colony.

### PpERF24 overexpression and *Physcomitrium patens* transformation

To generate *P. patens* lines overexpressing *PpERF24*, the Pp3c11_14690V3.1 coding sequence was amplified from gDNA using primers OXERF24 5′ fw (5′-AGGTAATGCAGTCGAGCATAC-3′) and OXERF24 3′ rv (5′-TCACCTGTCTAAGACAACC-3′). The PCR product was cloned into pGEM-T Easy vector (Promega). PpERF24 coding sequence was subcloned into *Eco*RI site of the pENTR2B vector (Gateway). The obtained pENTR2B:PpERF24 construct was sequenced by Sanger sequencing at Macrogen Inc. (Korea) and then transferred via LR clonase (Invitrogen) to a pTHUbi destination vector (Perroud et al., 2011). The obtained pTHUBi:PpERF24 construct was verified by Sanger sequencing at Macrogen Inc. (Korea). *P. patens* Gransden wild-type protoplast was transformed with pTHUBi-PpERF24 linearized with *Swa*I. Polyethyleneglycol-mediated transformation of protoplasts was performed according to Schaefer et al. (1991). Tissues of plants showing growth after hygromycin selection were harvested to analyze the incorporation of the transgene. Levels of *PpERF24* transcript accumulation of selected transformants were assayed by Northern blot analysis as described by Castro et al. (2016). For this, PpERF24 cDNA probe was labeled with [α32P]-dCTP using the Rediprime II Random Prime Labeling System kit (GE healthcare). DNA content of different PpERF24 overexpressing lines was measured by flow cytometry according to Castro et al. (2016). For this, 5,000 nuclei from each colony were analyzed. Haploid PpERF24 overexpressing lines were considered for further experiments.

### Subcellular localization of PpERF24-GFP

*PpERF24* coding sequence without the stop codon was amplified from *pMDC7:PpERF24* using primers mPpERF24BamHI_fw (5′-CTGGATGGATCCCCGAATTCACTAGTG-3′) and mPpERF24NotI_rv (5′-AATTCGCGGCCGCCTGTCTAAGA-3′) which contained restriction sites for *Bam*HI and *Not*I restriction enzymes, respectively. The corresponding fragment was cloned into *Bam*HI and *Not*I sites of the pENTR2B vector (Gateway) and transferred via LR clonase (Invitrogen) to vector pMDC83 (Curtis and Grossniklaus, 2003) containing the GFP coding sequence and 2 × 35 S promoter regulation. The obtained 2 × 35 S:PpERF24-GFP construct was sequenced by Sanger sequencing at Macrogen Inc. (Korea). For free GFP control, a pMDC83:ΔccdB construct, without the ccdB gene, was generated. For this, pMDC83 was digested with *Kpn*I, purified, and ligated. The obtained pMDC83:ΔccdB construct was sequenced by Sanger sequencing at Macrogen Inc. (Korea). The 2 × 35 S:PpERF24-GFP and pMDC83:ΔccdB constructs were introduced in *Agrobacterium tumefaciens* strain C58C1, and *Nicotiana tabacum* leaves were agroinfiltrated as described by Yang Y. et al. (2000). The fluorescence signals of the tobacco leaves were captured with a LSM Zeiss 800 confocal microscope (Carl Zeiss Microscopy) 2 days after agroinfiltration. Excitation laser lines 488 and 640 were used for GFP and chloroplast signal detection, respectively. The acquisition software used was ZEN Blue 2.6 (Carl Zeiss Microscopy). Image processing was done with ImageJ 1.52p (Schindelin et al., 2012).

### Sequence analysis of PpERF24 and comparison with other ethylene response factors and dehydration-responsive element-binding proteins

The analysis of conserved domains in PpERF24 protein was performed using the Simple Modular Architecture Research Tool (SMART) (Letunic et al., 2021) at http://smart.embl-heidelberg.de/. Secondary structure prediction was performed using JPred4 (Drozdetskiy et al., 2015) at http://www.compbio.dundee.ac.uk/jpred4/. The subcellular localization of PpERF24 proteins was predicted using Plant-mSubP (Sahu et al., 2020) at http://bioinfo.usu.edu/Plant-mSubP/. *PpERF24* orthologs and closest genes were identified by blast search on genomes available at phyotozome.org,^1^ including all moss genomes available at the moment (*P. patens*, *Ceratodon purpureus*, *Sphagnum fallax*, and *Sphagnum magellanicum*), the liverwort *Marchantia polymorpha*, the lycophyte *Selaginella moellendorffii*, and representative genomes of angiosperms (*A. thaliana*, *Glycine max*, *S. lycopersicum*, *Vitis vinifera*, and *O. sativa*); in all cases, the best blast hits were considered. To obtain homologous sequences in the hornwort *Anthoceros agrestis* Bonn genome, a tblastn analysis using PpERF24 as query sequence was performed at https://www.hornworts.uzh.ch/en.html (Li F. W. et al., 2020). To complete the search, an additional tblastn analysis using PpERF24 as query sequence was performed at NCBI,^2^ excluding *P. patens*; other species best blast hits obtained in this analysis with an *e*-value < 1.0E-10 were retrieved and the corresponding protein sequence downloaded. To further analyze these proteins and two *PpERF24* orthologs reported in *Funaria hygrometrica* (Kirbis et al., 2020), a blast search on the *P. patens* genome using these proteins as query sequences was performed at https://phytozome-next.jgi.doe.gov/ and NCBI, and sequence identity percentage with PpERF24 was obtained.

### Phylogenetic analysis

For phylogenetic analysis, multiple alignments of the full-length amino acid sequences derived from the AP2 domain of *P. patens*, *S. fallax*, *M. polymorpha*, *A. thaliana*, *O. sativa* subsp. *japonica*, and *S. lycopersicum* ERF family and moss proteins obtained in this work (Fh_17935, Fh_23703, CepurR40.9G091300.1.p, CepurR40.11G133100.1.p, TR55251, TR125756, DREB5-9, Sphfalx11G110500.1.p, and Sphmag11G113800.1.p) were performed with CLC Main Workbench ver. 21.0.4 (Qiagen). *P. patens*, *S. fallax*, *M. polymorpha*, *A. thaliana*, *O. sativa* subsp. *japonica*, and *S. lycopersicum* ERF family proteins sequences were retrieved from PlantTFDB v5 database (Jin et al., 2017). To obtain *A. agrestis* ERF family, 18 AP2/ERF proteins sequences reported by Li L. et al. (2020) were retrieved from *A. agrestis* Bonn genome at https://www.hornworts.uzh.ch/en.html, analyzed by SMART, and only those sequences fitting ERF family criteria (presence of one AP2 domain and absence of B3 domain) were considered. The AP2 domain of PpERF24 was used as a reference, and amino acids flanking it were trimmed from all proteins. These sequences were realigned, and the resulting alignment was used to create an unrooted phylogenetic tree by maximum likelihood using iqtree 1.3.11.1 software (Nguyen et al., 2015). The following parameters were used: ultrafast bootstrap (Minh et al., 2013) with 1,000 bootstrap replicates and standard model selection followed by tree construction. The best-fit model LG + G4 was chosen according to Bayesian information criterion. The phylogenetic tree was visualized with iTOL6.5.2 (Letunic and Bork, 2021). The AP2 domains of the different clades were retrieved, and sequences logos for conserved motifs were obtained from multiple sequence alignments of AP2 domains using CLC Main Workbench ver. 21.0.4 (Qiagen). Logos for *A. thaliana* ERF and DREB subfamilies, *P. patens* ERF and DREB subfamilies, mosses-specific clade, and PpERF24 subclade were generated.

### Cell death measurements

For cell death measurements, *P. patens* colonies treated with CF were incubated with 0.1% Evans blue for 1 h, whereas *P. patens* colonies inoculated with fungi and *P. irregulare* were incubated with 0.05% Evans blue for 2 h. After incubation, tissues were washed four times with deionized water to remove excess and unbound dye. Dye bound to dead cells was solubilized in 50% methanol with 1% SDS for 45 min at 60°C, and the absorbance was measured at 600 nm (Levine et al., 1994; Oliver et al., 2009). Samples were dried at 60°C for 16 h, and the results were expressed as Abs600 nm/mg dry weight (DW). Eight samples containing four colonies were analyzed for each genotype and treatment, and each experiment was repeated at least three times. Data from each experiment were tested for normality and further compared using a two-way ANOVA where *p*-values < 0.05 were considered statistically significant. Statistical analysis was performed with GraphPad Prism software ver. 8.0.2.

### Quantification of pathogen biomass by quantitative PCR

Pathogen biomass in *P. patens* tissues inoculated with *B. cinerea* or *P. irregulare* at 24 hpi and with *C. gloeosporioides* at 72 hpi was quantified by quantitative PCR (qPCR). Sixteen *P. patens*-infected colonies were used for each biological replicate. Samples were frozen in liquid nitrogen, and DNA was extracted with DNeasy kit (Qiagen) and quantified using NanoDrop spectrophotometer. qPCR was performed using primers designed for the ubiquitin gene of *P. patens* (Ubi2), ITS for *B. cinerea* and *P. irregulare*, and CFP12895 for *C. gloeosporioides* (Pathompitaknukul et al., 2020; Supplementary Table 1). qPCR was performed in an Applied Biosystems QuantStudio 3 thermocycler using the QuantiNova Probe SYBR Green PCR Kit (Qiagen). Mix proportions and cycling parameters were used as described in the manufacturer’s instructions. For standard curves, a threefold serial dilution of genomic DNA from *P. patens*, *B. cinerea*, *C. gloeosporioides*, and *P. irregulare* with known concentrations (3, 1, 0.33, 0.11, and 0.037 ng) was used. Each pathogen and *P. patens* DNA standard curves were generated by plotting the CT values of the threefold serial dilutions vs. the logarithm of DNA concentration. The resulting regression equations were used to calculate pathogen and plant DNA in each sample, and DNA rate was reported (expressed as ng pathogen/ng *P. patens*). Each data point is the mean value of three biological replicates. The Student’s *t*-test was applied to all qPCR data, and values of *p* ≤ 0.05 were considered statistically significant.

### RNA extraction, cDNA library preparation, and sequencing

For RNA extraction, three independent biological replicates of tissue were harvested from wild-type and PpERF24-OX-4 plants treated with water (control) or *B. cinerea* at 24 hpi, immediately frozen in liquid nitrogen, and stored at –80°C. Frozen samples were pulverized with a mortar and pestle, and total RNA was extracted using the RNeasy Plant Mini Kit (Qiagen), including an RNase-Free DNase treatment in column (Qiagen), according to the manufacturer’s protocol. RNA quality control, library preparation, and sequencing were performed at Macrogen Inc. (Korea). RNA integrity was checked before library preparation using an Agilent Technologies 2100 Bioanalyzer (Agilent Technologies). Libraries for each biological replicate were prepared for paired-end sequencing by TruSeq Stranded Total RNA LT Sample Prep Kit (Plant) with 1 μg input RNA, following the TruSeq Stranded Total RNA Sample Prep Guide, Part # 15031048 Rev. E. Sequencing was performed on Illumina platform by Macrogen Inc. (Korea) to generate paired-end 101 bp reads, obtaining 46–66 M reads per sample with Q20 > 97, 85%.

### RNA-seq processing and differential expression analysis

RNA-seq processing steps were done through Galaxy platform (Afgan et al., 2018) at https://usegalaxy.org/, following Reboledo et al. (2021) pipeline. Trimmed reads were mapped to reference genomes of *P. patens* using Hisat2 software (Kim et al., 2015). All reads were mapped first against *P. patens* organelle genomes and rRNA sequences (mitochondrial NC_007945.1; chloroplast NC_005087.1; ribosomal HM751653.1, X80986.1, and X98013.1). The remaining reads were mapped against *P. patens* nuclear genome v3 (Lang et al., 2018), using Ppatens_318_v3.fa as the reference genome file and Ppatens_318_v3.3.gene_exons.gff3 as a reference file for gene models,^3^ and concordant mapped read pairs were retained. The BAM files with reads mapped in proper pairs were obtained with Samtools View software ver. 1.9 and then sorted by name with Samtools Sort software ver. 2.0.3 (Li et al., 2009).

Reads were counted by the FeatureCounts software ver. 1.6.4 (Liao et al., 2014) as in Reboledo et al. (2021). Differential expression analyses were performed using edgeR software ver. 3.24.1 (Robinson et al., 2010; Liu et al., 2015) with *p*-value adjusted threshold 0.05, *p*-value adjusted method Benjamini and Hochberg (1995), and minimum log2 fold change 1. For comparison, counts were normalized with TMM method. Low-expressed genes were filtered for count values ≥ 5 in three or more samples. In this study, a false discovery rate (FDR) ≤ 0.05 was used to determine significant differentially expressed genes (DEGs) between samples, and expression values were represented by log2 ratio. Hierarchical clustering and heatmaps plots of expressed genes were performed on log2 fold change expression values using the R package “gplots” ver. 3.1.0.

### Gene ontology enrichment analysis

Gene ontology (GO) and functional annotations were assigned based on *P. patens* v3.3 gene models (see text footnote 3) and Blast2GO ver. 5.2.5 software (Götz et al., 2008), through Blast and Interpro searches of homologs and protein domains (Reboledo et al., 2021). DEG functional enrichment analysis was performed using OmicsBox tools.^4^ GO terms with a FDR ≤ 0.05 were considered for our analysis.

### Gene expression analysis

RNA extraction of tissues from four independent biological replicates of wild-type, PpERF24-OX-2 and PpERF24-OX-4 control (water) and 24 h after inoculation with *B. cinerea* or *C. gloeosporioides*, was performed with RNeasy Plant Mini Kit (Qiagen). The expression level of selected defense-related genes was analyzed by RT-qPCR. After RNase-Free DNase treatment, cDNA was generated from 1 μg of RNA using RevertAid Reverse Transcriptase (Thermo Scientific) and oligo (dT) according to the manufacturer’s protocol. RT-qPCR was performed in an Applied Biosystems QuantStudio 3 thermocycler using the QuantiNova Probe SYBR Green PCR Kit (Qiagen); mix proportions and cycling parameters were used as described in the manufacturer’s instructions. Relative expression of each gene was normalized to the quantity of constitutively expressed Ubi2 gene (Le Bail et al., 2013), using the 2^–ΔΔCt^ method (Livak and Schmittgen, 2001). Gene expression in control or pathogen-inoculated tissues of the different genotypes was expressed relative to the corresponding control or pathogen-inoculated samples of the wild-type. Each data point is the mean value of four biological replicates. The Student’s *t*-test was performed to determine the significance for quantitative gene expression analysis using GraphPad Prism software ver. 8.0.2. *P*-values ≤ 0.05 were considered statistically significant. Primer pairs used for qPCR analyses are provided in Supplementary Table 1.

## Results

### *PpERF24* is a moss-specific pathogen-inducible gene

**FIGURE 1:**
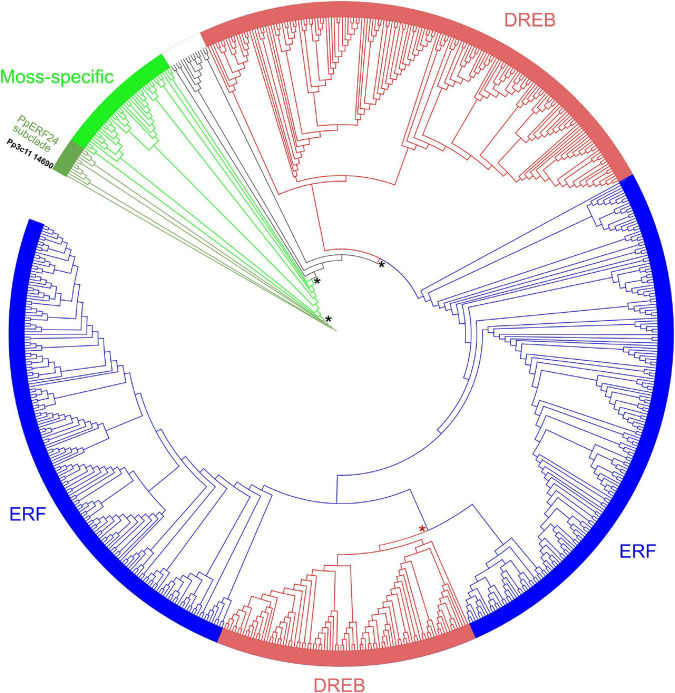
Unrooted phylogenetic tree of AP2 domains of moss, liverwort, hornwort, and angiosperms. Unrooted maximum likelihood phylogenetic tree was visualized with iTOL. Subfamilies are indicated with different colors: ERFs (blue), DREBs (red), and moss-specific proteins (green). The moss-specific clade, the PpERF24 subclade, ERFs, and DREBs clades are supported by bootstrap values ≥ 95 (black asterisk). A bootstrap of 92 separate ERFs and DREBs (red asterisk). Pp3c11_14690 (PpERF24) is highlighted in black. See Supplementary Table 4 for complete information.

In a previous study, we identified a member of the AP2/ERF superfamily (Pp3c11_14690) that was induced during treatment with elicitors of *P.c. carotovorum* (Alvarez et al., 2016). This gene has an open reading frame of 534 nucleotides and encodes a protein of 177 amino acids referred as *PpERF24* according to AP2/ERF members at PlantTFDB v5.0 database (Supplementary Table 2). The predicted AP2 domain consists of 63 amino acids with three-stranded antiparallel β-sheet (β1–β3) and an α-helix according to SMART. We searched for proteins with high identity to PpERF24 at NCBI and Phytozome database and identified three *P. patens* genes that covered amino acids 19 or 20—177 and exhibited 58 and 67% of identity, including Pp3c7_10780, Pp3c2_25760, and Pp3c1_14230 (Supplementary Table 3). Other *P. patens* genes showed 69% identity only in the AP2 domain (39–99 aa). Putative orthologs were only identified in other moss species; CepurR40.9G091300 of *C. purpureus* shared 68% identity with PpERF24 in amino acids 38–177, and TR55251_c0_g3_i1 of *Bryum argenteum* shared 66% identity in amino acids 31–177 (Supplementary Table 3). Two additional orthologs were identified in the moss *F. hygrometrica* (Fh_17935 and Fh_23703) (Kirbis et al., 2020). Interestingly, the liverwort *M. polymorpha*, the hornwort *Anthoceros agrestis*, the lycophyte *S. moellendorffii*, and angiosperms did not have orthologs (Supplementary Table 3). To further analyze the characteristics of these proteins in the ERF family of *P. patens* (143 ERF + DREB), the deduced amino acid sequences of the AP2 domains were obtained from the PlantTFDB v5.0 database and aligned with ERF family members of *S. fallax* (115), *M. polymorpha* (23), *A. thaliana* (123), *O. sativa* subsp. *japonica* (138), and *S. lycopersicum* (136). Additionally, 13 *A. agrestis* ERF members and the moss proteins identified by blast analysis in Supplementary Table 3 were included in the alignment. An unrooted phylogenetic tree was constructed, showing well-supported clades that separated a moss-specific clade with *P. patens*, *S. fallax*, and other moss proteins, and several clades including *A. thaliana* DREBs and ERFs groups described by Nakano et al. (2006), together with *P. patens*, *S. fallax*, *M. polymorpha*, *A. agrestis*. *O. sativa*, and *S. lycopersicum* proteins (Figure 1). A small number of ERFs of different species, 13 in total, containing members of *P. patens* and *S. fallax* and one member of *A. agrestis*, *A. thaliana*, *O. sativa*, and *S. lycopersicum* were not included in any of these clades. Based on the tree and the ERF family classification in *A. thaliana*, *P. patens* has 23 moss-specific proteins, 56 DREBs, 59 ERFs, and five proteins that do not belong to any of these clades (Supplementary Table 4). Interestingly, PpERF24 was included in the moss-specific clade, forming a subclade with the three *P. patens* homologs Pp3c7_10780, Pp3c2_25760, and Pp3c1 14230, the orthologs CepurR40.9G091300, TR55251, Fh_23703, Fh_17935, and four additional sequences: Sphmag11G113800, Sphfalx11G110500 (corresponds to Sphfalx0096s0061 according to PlantTFDB v5.0 database), Sphfalx0299s0005, and Sphfalx0027s0138.

**FIGURE 2:**
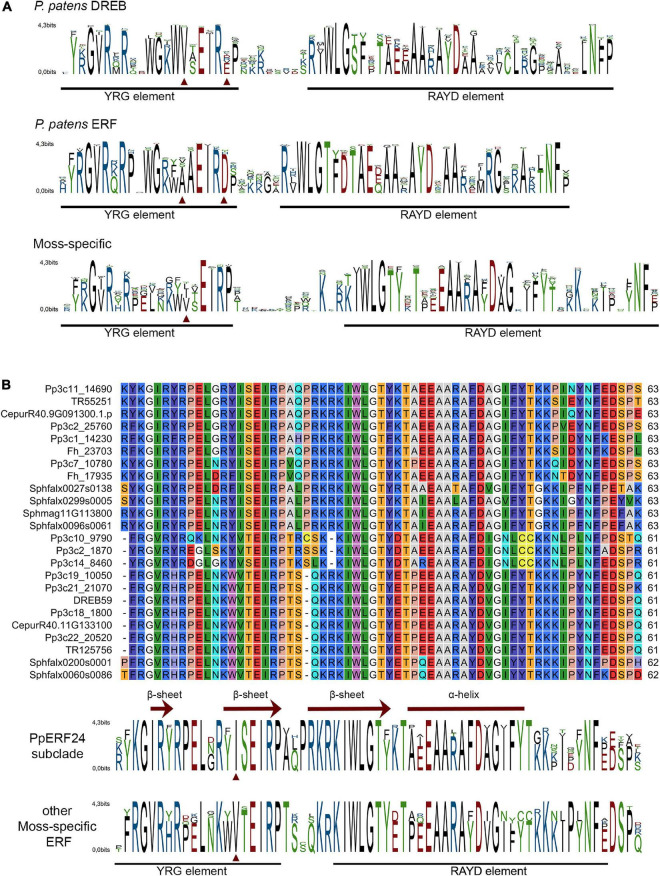
Conserved motifs in the AP2 domain of mosses ERF family clades **(A)**. Sequence logos of AP2 domain from *P. patens* DREB clade, *P. patens* ERF clade, and moss-specific clades. **(B)** Alignment of PpERF24 and several AP2 domains of the moss-specific clade and corresponding sequence logos. Conserved YRG and RAYD elements are indicated with a black line, and conserved amino acids 14 and 19 for members of the DREB and ERF clades and 15 for PpERF24 and moss-specific AP2 domains are marked with red arrows. The β-sheets and α-helix predicted for PpERF24 are shown.

Multiple alignments of the AP2 domain of *P. patens* and *A. thaliana* members of the DREB and ERF clades showed highly conserved signatures in the YRG and RAYD elements (Supplementary Figures 1–4). The motifs YRG or YKG and WLG involved in DNA binding were present in most AP2 domains of *P. patens* ERFs and DREBs (Figure 2A). The AYD signature of the RAYD motif involved in protein–protein interactions and DNA binding was highly conserved in *P. patens* ERFs and DREBs, while the R residue changed to other amino acids in several members of both species (Figure 2A). Furthermore, V14 and E19 that are typical of DREBs (Zhuang et al., 2008; Duan et al., 2013) were present in most *P. patens* DREBs (Figure 2A and Supplementary Figure 1). Similarly, A14 and D19 that define ERFs were found in most *P. patens* ERFs (Figure 2A and Supplementary Figure 2). In contrast, alignment of the AP2 domains of the moss-specific clade showed less conserved signatures in the YRG and RAYD elements (Figure 2A and Supplementary Figure 5). For example, a high number of moss-specific AP2 domains have an additional E at position 10 (E10), resulting in an RxRPELG/NK/R signature, which changed in ERF and DREB to RxRxWGK/R. Moreover, moss-specific AP2 domains lack the amino acid at position 19, and at the end of the YRG element, they exhibit an EIRP motif instead of EIREP in DREBs or EIRVP in ERFs. Thus, the conserved amino acid at position 14 changed to 15 and was in most moss-specific AP2 domains a V15 or an isoleucine (I15), and they exhibited a proline at position 20 (P20) (Figure 2A). Moreover, whereas the WLG motif was conserved in almost all moss-specific AP2 domains, other motifs such as FKG and RAFD were more frequent in moss-specific proteins than *P. patens* and *A. thaliana* DREBs and ERFs. When we looked at the signatures of the 12 AP2 domains that did not group with any of these clades, we observed that they have differences in some of the conserved signature of the YRG and RAYD elements (Supplementary Figure 6). For example, Pp3c17_13620 and Pp3c1_32440 have a YAYD motif, and Sc2ySwM_362.93 (*A. agrestis*) and the three AP2 domains of angiosperms present in this group (AT4G13040, Solyc09g059510, LOC_Os02g29550) exhibited a MRG and a RLYD motif and did not have the conserved amino acids at position 14 and 19. When we looked in more detail to sequence alignment of the PpERF24 subclade and several AP2 domains of the moss-specific clade, we found clear differences in amino acid composition in the YRG and RAYD elements of both groups (Figure 2B). The most remarkable change is the presence of an I15 in the PpERF24 subclade and a V15 in the rest of the proteins. Changes in other signatures include: YKGIR and RYIS in the YRG element of the PpERF24 subclade that is converted to FRGVR and KWVT in the other moss-specific proteins. Additionally, members of the PpERF24 subclade have a K in the RAYD element. Interestingly, all *P. patens* members of the PpERF24 subclade are induced by the fungal pathogens *B. cinerea* and *C. gloeosporioides* (Reboledo et al., 2022), suggesting a possible role in moss defense against biotic stress. Based on these findings, we further characterized PpERF24 and evaluated its role in moss defenses against pathogen infection.

### Subcellular localization of PpERF24

According to subcellular localization prediction using Plant-mSubP (Sahu et al., 2020), PpERF24 goes to the nucleus. To confirm this prediction, the intracellular localization of PpERF24 was determined by transient expression of the PpERF24:GFP fusion protein in *N. tabacum* leaves (Supplementary Figure 7). Consistent with the function of a TF, confocal microscopy showed that PpERF24:GFP fusion protein was located in the nucleus and the fluorescent signals of the control were observed in whole cells.

### Overexpression of *PpERF24* contributes to defenses against fungal pathogens in *Physcomitrium patens*

**FIGURE 3:**
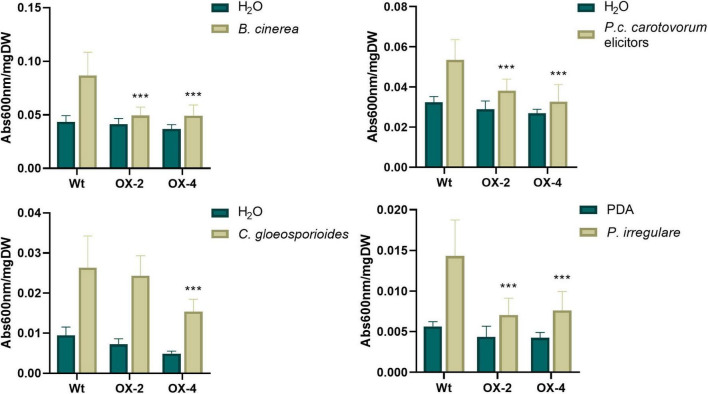
Cell death measurement in wild-type and PpERF24 overexpressing plants. Measurement of cell death by Evans blue staining 24 h after inoculation of wild type, PpERF24-OX-2 (OX2), and PpERF24-OX-4 (OX4) moss colonies with *B. cinerea, P. irregulare*, and treatment with elicitors of *P.c. carotovorum*. Cell death measurement for plant tissues inoculated with *C. gloeosporioides* was performed at 72 hpi. Data were expressed as the absorbance (Abs) at 600 nm per milligram of dry weight (DW). Values are means with standard deviations of eight independent replicate moss samples. Experiments were repeated thrice with similar results. Asterisks indicate a statistically significant difference between the wild-type and overexpressing PpERF24 plants [two-way ANOVA test, with Tukey’s honest significant difference test as *post hoc* test using *P* ≤ 0.001 (***)].

*P. patens* lines overexpressing *PpERF24* were generated, and two transformants designated PpERF24-OX-2 and PpERF24-OX-4 exhibiting high constitutive levels of *PpERF24* were selected for further studies (Supplementary Figure 8A). RT-qPCR analysis showed that expression levels of PpERF24 in PpERF24-OX-2 and PpERF24-OX-4 PpERF24 increased more than 300 and 900 times compared to wild-type plants (Supplementary Figure 8B). No variation in the phenotype of overexpressing lines compared to wild-type plants was observed during gametophytic growth under normal growth conditions (Supplementary Figure 8C). The possible role of PpERF24 during defense responses against pathogens was evaluated by inoculating moss colonies with *B. cinerea*, *C. gloeosporioides*, *P. irregulare*, or treated with elicitors of *P.c. carotovorum*, and measuring cellular damage. All these pathogens are necrotrophic, and although *C. gloeosporioides* is considered a hemibiotrophic pathogen, the biotrophic phase has not been detected in *P. patens* and some angiosperms (Reboledo et al., 2015). Symptom development between genotypes did not show visible differences after treatments with pathogens or bacterial elicitors (Supplementary Figure 9). When cell death was measured, we observed that cellular damage caused by *C. gloeosporioides* was lower compared to *B. cinerea* and elicitors of *P.c. carotovorum* in the three genotypes probably due to differences in the infection process (Figure 3). Interestingly, cell death decreased significantly in PpERF24-OX-2 and PpERF24-OX-4 compared to wild-type tissues, after inoculation with *B. cinerea*, *P. irregulare*, and treatment with elicitor of *P.c. carotovorum*, while only the line with the highest *PpERF24* expression levels (PpERF24-OX-4) exhibited less cellular damage against *C. gloeosporioides* (Figure 3). Moreover, while no significant differences were observed with *P. irregulare*, qPCR measurement of *B. cinerea* and *C. gloeosporioides* DNA confirmed that less biomass of *B. cinerea* was present in the two overexpressing lines and that *C. gloeosporioides* biomass decreased only in PpERF24-OX-4 compared to the other two genotypes (Supplementary Figure 10). These findings indicate that PpERF24 contributed to defenses against fungal pathogens, evidenced by less cellular damage and less pathogen biomass.

**FIGURE 4:**
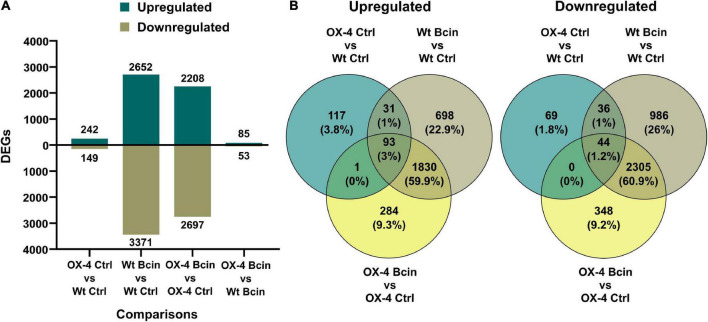
Differentially expressed genes of wild type and PpERF24-OX-4 overexpressing line during control treatment and *B. cinerea* infection. **(A)** Number of differentially expressed genes (DEGs), up- and downregulated, in wild type vs. PpERF24-OX-4 (OX4) tissues treated with water (Ctrl), and wild type (Wt) and PpERF24-OX-4 tissues inoculated with *B. cinerea* (Bcin) vs. water-treated tissues at 24 hpi. **(B)** Venn diagram of *B. cinerea*-responsive *P. patens* genes in wild-type and PpERF24-OX-4 plants and PpERF24-OX-4 vs. wild-type control tissues. Genes were considered as DEGs when | log2 FC| ≥ 1 and FDR ≤ 0.05.

To get more insights into the function of PpERF24 in defense mechanisms, we performed a transcriptional profiling analysis of wild-type and PpERF24-OX-4 plants after 24 h of control treatment or *B. cinerea* inoculation. RNA-seq generated a total of 268 million reads that mapped to the nuclear genome (Supplementary Table 5). A total of 391 differentially expressed genes (DEGs) were identified when PpERF24-OX-4 control plants were compared with control wild-type plants (242 were upregulated and 149 were downregulated) (Figure 4 and Supplementary Table 6). Interestingly, 51% (124) of these upregulated DEGs were also induced during *B. cinerea* infection in wild-type plants, and 54% (80) of the downregulated DEGs were also repressed in wild-type infected plants. In total, 2,652 and 3,371 genes were induced and repressed, respectively, in wild-type *B. cinerea*-inoculated plants compared to control plants. Similarly, 2,208 induced and 2,697 repressed genes were present in the comparison between *B. cinerea*-inoculated PpERF24-OX-4 and the corresponding control tissue (Figure 4A). Comparison between PpERF24-OX-4 and wild-type plants inoculated with *B. cinerea* resulted only in 85 upregulated and 53 downregulated genes (Figure 4A). Furthermore, 1,923 genes were commonly upregulated during *B. cinerea* inoculation in PpERF24-OX-4 and wild-type plants compared to the corresponding controls (Figure 4B). Similarly, a high number of DEGs (2349) were downregulated in both genotypes when *B. cinerea* and control treatments were compared (Figure 4B).

Enrichment of GO terms was performed to identify the biological processes (BPs) and molecular functions (MFs) mostly affected by *B. cinerea* infection in wild type and PpERF24-OX-4 (Supplementary Figure 11). A high number of the top 15 significantly enriched GO terms for upregulated and downregulated genes were similar in *B. cinerea*-infected wild-type and PpERF24-OX-4 plants. For upregulated DEGs, enriched GO BP terms included phenylpropanoid metabolic process, secondary metabolic process, cinnamic acid biosynthetic process, response to fungus, defense response, response to oxygen-containing compound, and defense response to fungus. Similarly, enriched MF GOs were related to naringenin-chalcone synthase activity, DNA-binding transcription factor activity, transcription regulator activity, phenylalanine ammonia-lyase activity, heme binding, ammonia-lyase activity, catalytic activity, tetrapyrrole binding, carbon-nitrogen lyase activity, oxidoreductase activity, active transmembrane transporter activity, and nutrient reservoir activity. For downregulated DEGs, enriched GOs included terms related to photosynthesis, responses to different types of lights, and response to abiotic stimulus (Supplementary Figure 11). No enriched GO terms were detected for the comparison between PpERF24-OX-4 and wild-type plants inoculated with *B. cinerea*. However, control PpERF24-OX-4 vs. control wild-type plants showed only enriched GO terms in upregulated genes, including DNA-binding transcription factor activity and transcription regulator activity.

### PpERF24 overexpressing lines exhibited induced expression of defense-related genes

**FIGURE 5:**
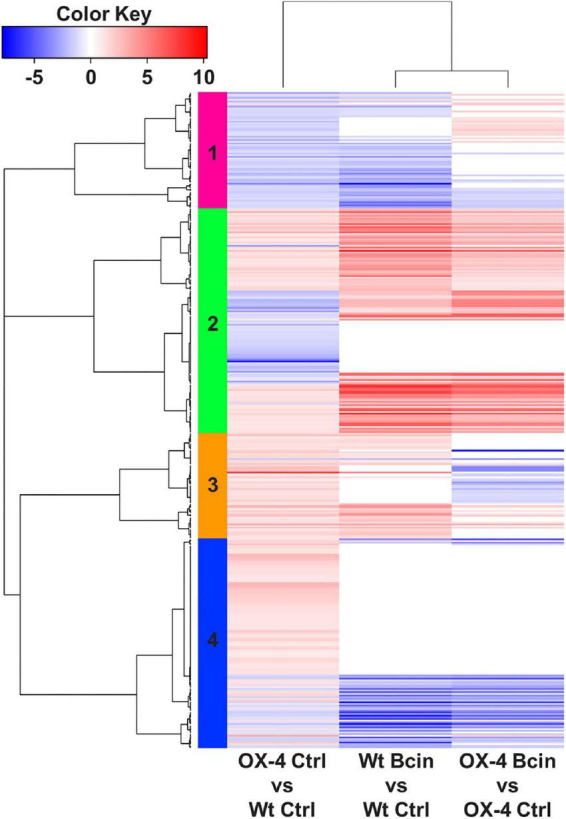
Heatmap of hierarchical clustering of wild type and PpERF24-OX-4 overexpressing line during control treatment and *B. cinerea* infection. DEGs correspond to the 391 DEGs of PpERF24-OX-4 control vs. wild-type control plants. Selected DEGs had | log2 FC| ≥ 1 and FDR ≤ 0.05. See Supplementary Table 7 for complete information.

**FIGURE 6:**
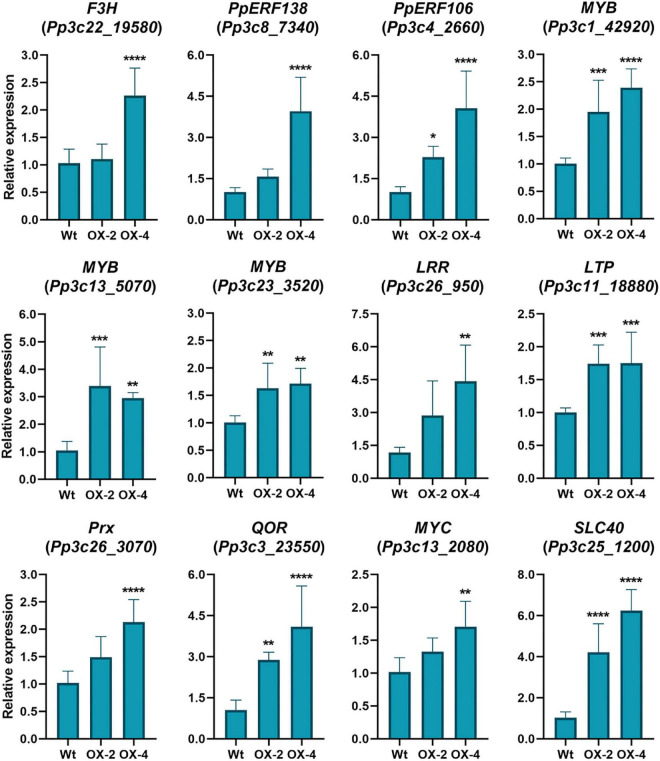
Expression levels of defense genes in control wild-type and PpERF24 overexpressing plants. Transcript levels in control PpERF24 overexpressing lines were expressed relative to the corresponding levels in control wild-type samples, using Ubi2 as reference gene. Results are reported as means ± standard deviation (SD) of four samples for each treatment. Asterisks indicate a statistically significant difference between wild-type and overexpressing plants (Student’s *t*-test, *P ≤ 0.05; **P ≤ 0.01; ***P < 0.001; ****P < 0.0001).

Since PpERF24-OX-4 compared to wild type under control treatment showed differential expression of genes with possible roles in defense, such as TFs, compared to wild-type plants, we first focused on the group of 391 DEGs. Hierarchical clustering identified a group of upregulated DEGs in the comparison of PpERF24-OX-4 control vs. wild-type control, which did not show differential expression during *B. cinerea* inoculation in PpERF-OX-4 and wild-type plants when compared to their respective controls (Figure 5, clusters 2, 3, and 4). This result could be due to higher or less expression levels in PpERF-OX-4 that did not increase or decrease significantly during *B. cinerea* inoculation and were therefore not considered as DEGs. However, several groups of genes that showed increased expression in PpERF-OX-4 control samples compared to wild-type control samples were also induced by *B. cinerea* in both genotypes (clusters 2 and 3) (Figure 5). Within these upregulated DEGs, genes encoded seven ERFs (Pp3c10_20000, Pp3c13_4270, Pp3c3_6420, Pp3c4_2660, Pp3c4_2680, Pp3c6_26080, and Pp3c7_10780), four MYBs including TT2 (Pp3c16_10020, Pp3c24_7590, Pp3c1_42920, and Pp3c13_5070), one PLATZ (Pp3c15_23190), and other proteins involved in defense against biotic stress, including chitin elicitor receptor kinase 1 (CERK1; Pp3c5_4120), chitinase (Pp3c8_22560), respiratory burst oxidase (RBOH; Pp3c24_2840), beta-1,3 glucanase (Pp3c3_18870), cupin (Pp3c7_25050), peroxidases (Pp3c17_6050 and Pp3c26_3070), and flavonoid 3′-hydroxylases (F3H) (Pp3c25_15450 and Pp3c22_19580) (Supplementary Table 7). Furthermore, genes encoding a lipase (Pp3c11_3620), an allene oxide cyclase (AOC; Pp3c2_24500), a 12-oxophytodienoic acid reductase (OPR; Pp3c3_2130), and MYC (Pp3c13_2080) involved in the jasmonate pathway were upregulated. Additionally, in PpERF24-OX-4 genes involved in cell wall remodeling and integrity maintenance were upregulated compared to wild-type plants under control conditions, including xyloglucan xylosyltransferases (Pp3c4_6640 and Pp3c9_7720), protein trichome birefringence (Pp3c7_1960), mannan polymerase II complex (Pp3c7_7810), galacturonosyl transferases (Pp3c5_28420 and Pp3c16_25090), and cobra-like protein (Pp3c27_3700). Increased expression of several genes with functions related to plant defense against pathogens in control overexpressing lines compared to control wild-type plants was confirmed with RT-qPCR (Figure 6). The results show that genes encoding F3H (Pp3c22_19580), ERF138 (Pp3c8_7340), ERF106 (Pp3c4_2660), three MYBs (Pp3c1_42920, Pp3c13_5070, and Pp3c23_3520), a leucine-rich repeat (LRR) receptor-like kinase (Pp3c26_950), a lipid transfer protein (LTP) (Pp3c11_18880), a peroxidase (Prx) (Pp3c26_3070), a NADPH2:quinone reductase (QOR) (Pp3c3_23550), MYC (Pp3c13_2080), and a solute carrier family 40 (SLC40: Pp3c25_1200) were induced in PpERF24-OX-4 or in both overexpressing lines compared to wild-type tissues. This result confirmed that both lines have increased expression of defense genes. Five genes were only induced in PpERF24-OX-4, while seven genes were induced in PpERF24-OX-4 and PpERF24-OX-2, which is consistent with higher expression levels of PpERF24 in PpERF24-OX-4.

**FIGURE 7:**
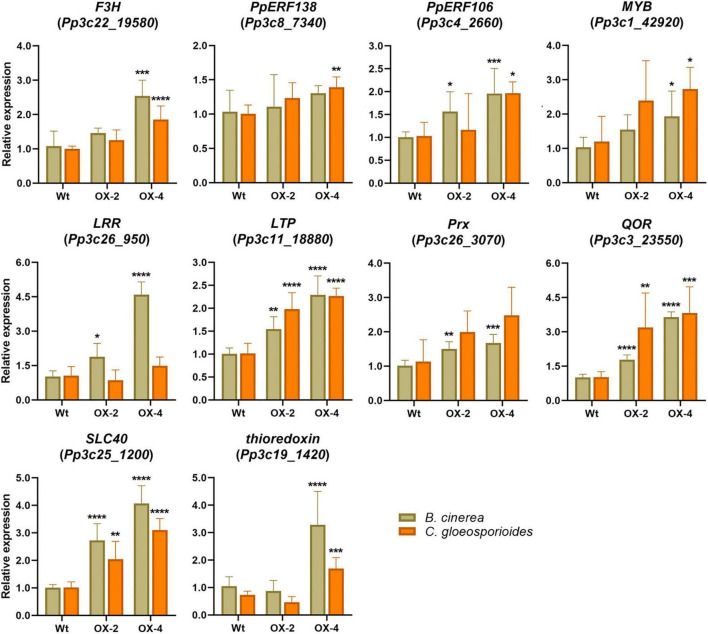
Expression levels of defense genes in fungal-inoculated wild-type and PpERF24 overexpressing plants. Transcript levels in PpERF24 overexpressing lines inoculated with *B. cinerea* or *C. gloeosporioides* at 24 hpi were expressed relative to the corresponding levels in pathogen-inoculated wild-type plants, using Ubi2 as reference gene. Results are reported as means ± standard deviation (SD) of four samples for each treatment. Asterisks indicate a statistically significant difference between wild-type and overexpressing plants (Student’s *t*-test, *P ≤ 0.05; **P ≤ 0.01; ***P < 0.001; ****P < 0.0001).

When we looked in more detail to upregulated DEGs in the comparison between PpERF24-OX-4 and wild-type plants inoculated with *B. cinerea*, we identified several genes with possible roles in defense that showed higher expression levels in the overexpressing lines compared to wild-type plants. This is the case for genes encoding protein kinase (Pp3c17_14330), LRR receptor-like kinase (Pp3c26_950), receptor protein-tyrosine kinase (Pp3c10_19910), thioredoxin (Pp3c19_1420), F3H (Pp3c22_19580), MYB (Pp3c1_42920), ERF103 (Pp3c4_24710), RAV2 (Pp3c2_23660), and auxin influx carrier (Pp3c12_5490), among others. Interestingly, Pp3c17_12450 and Pp3c17_4880 that were considered as orphan genes according to Reboledo et al. (2021) exhibited higher expression levels in PpERF24-OX-4 compared to the wild type. In addition, genes encoding for a cysteine-rich secretory protein (Pp3c18_21170), a TIR domain-containing protein (Pp3c17_15630), a LRR receptor-like kinase ERECTA (Pp3c7_3320) were only upregulated in PpERF24-OX-4-inoculated plants and not in wild-type plants. To further confirm that PpERF24 contributes to defense against fungal pathogens, we analyzed the expression levels of defense genes in response to *B. cinerea* and *C. gloeosporioides* in wild-type and overexpressing plants (Figure 7). The results confirmed increased gene expression in one or both PpERF24 overexpressing lines in response to one or both pathogens compared to the wild type, including *F3H*, *ERF106*, *MYB* (Pp3c1_42920), *LRR receptor-like kinase*, *LTP*, *Prx*, *QOR*, *SLC40*, and a thioredoxin (Pp3c19_1420). Taken together, these findings suggest that PpERF24 positively regulates the expression of defense genes, leading to increased resistance to fungal pathogens.

## Discussion

Land plants, including mosses, have evolved different defense mechanisms to protect themselves against invading pathogens and abiotic stresses. One of these defense mechanisms includes the activation of AP2/ERF TFs (Otero-Blanca et al., 2021; Reboledo et al., 2022). In *P. patens*, at least 161 AP2/ERF genes have been identified and a high proportion of them responds to different types of abiotic stresses and biotic interactions (Reboledo et al., 2022). ERF family is divided into ERF and DREB subfamilies based on the sequence similarity of their AP2 domain, including conserved amino acids within the YRG element that are important for DNA binding (Allen et al., 1998; Sakuma et al., 2002). This includes amino acid 14 and 19, A14 and D19 for ERFs, and the V14 and E19 for DREBs, which bind to promoter regions of the target genes and modulate their expression (Allen et al., 1998; Sakuma et al., 2002). According to our phylogenetic tree and *A. thaliana* classification, 23 *P. patens* proteins belong to a moss-specific clade, 56 *P. patens* DREBs and 59 *P. patens* ERFs are grouped with *A. thaliana* DREB and ERF subfamilies, and five proteins did not group with any of these clades. Interestingly, while most *P. patens* and *A. thaliana* proteins in the ERF and DREB clades have the conserved amino acids at positions 14 and 19, resulting in VXEIREP and AAEIRDP motifs at the end of the YRG element, PpERF24 and other *P. patens* proteins within the moss-specific clade have a conserved V15 or I15 within a VXEIRP motif and a P at position 20. Consistently, several members of a *B. argenteum*-specific ERF clade have a V15 and a P20 in the AP2 domain (Li et al., 2018). It has been previously shown that while DREB V14 is generally conserved in different plant species, several angiosperms have a leucine or a valine instead of E19 (Dubouzet et al., 2003; Wang et al., 2011), suggesting that the function of the 14th amino acid is likely more important than the 19th for specific DNA-binding activity (Wang et al., 2011). Here, we show that the amino acid at position 19 (E, D, or other) is absent in the moss-specific AP2 domains. Moreover, in *P. patens*, V15 was conserved in 10 members (43%) of the moss-specific clade, while other members of this clade have I15 (five proteins), or other amino acids in that position (eight proteins). Remarkably, the signatures YKGIR and RYIS that includes I15 in the YRG element separated PpERF24 subclade from the rest of the moss-specific proteins. How moss-specific and PpERF24 subclade signatures within the YRG element affect DNA binding to specific elements and subsequent gene expression need further investigation. Interestingly, all *P. patens* members of the PpERF24 subclade are induced by the pathogens *B. cinerea* and *C. gloeosporioides* (Reboledo et al., 2022), suggesting a possible role in moss defense.

Overexpression of *PpERF24* generated moss plants that exhibited less cellular damage to *B. cinerea*, *P. irregulare*, and treatment with elicitors of *P. c. carotovorum* compared to wild-type plants, while the line that exhibited the highest expression level of PpERF24 (PpERF24-OX-4) also showed less cell death with *C. gloeosporioides*. Interestingly, *B. cinerea* biomass decreased in both overexpressing lines, while *C. gloeosporioides* biomass decreased only in PpERF24-OX-4, which is consistent with the levels of cellular damage in the different genotypes. Previous studies have shown that overexpression of ERF1 confers resistance in *A. thaliana* to several fungi, including *B. cinerea* (Berrocal-Lobo et al., 2002). Moreover, overexpression of ERF TF ORA59 (octadecanoid-responsive Arabidopsis) increases resistance against *P. c. carotovorum*, while mutant ora59 plants were more susceptible to this pathogen (Kim et al., 2018). ORA59 interacts in the nucleus with another ERF (RAP2.3) and plays a positive role in ethylene-regulated responses (Kim et al., 2018).

Transcriptional profiling and GO enrichment analysis of PpERF24-OX-4 and wild-type plants revealed that in response to *B. cinerea* similar categories of genes were upregulated. These include genes encoding proteins of important defense pathways and proteins with diverse roles in defense, such as receptors involved in the perception of the pathogen, kinases and calcium signaling, transcriptional regulation, cell-wall-associated defenses, phenylpropanoid pathway, and hormonal pathways including the jasmonate pathway. Furthermore, genes encoding PR proteins with antimicrobial activities were upregulated in response to *B. cinerea* inoculation in wild type and PpERF24-OX-4, including *PR-1* (small cysteine-rich secreted protein), *PR-2* (β-1,3 glucanases), *PR-3* (chitinase), *PR-5* (thaumatin), *PR-9* (peroxidases), and *PR-10* (Bet v I). Similarly, photosynthesis-related processes were downregulated in both genotypes after fungal infection, which could be related to browning and breakdown of chloroplasts (Ponce de León et al., 2007), and reallocation of nitrogen resources for the synthesis of proteins involved in defense as has been observed in angiosperms (Windram et al., 2012). In total, 391 genes were differentially expressed in PpERF24-OX-4 compared to wild type under control conditions. Interestingly, approximately 50% of these upregulated genes were also upregulated during *B. cinerea* inoculation in wild-type plants, suggesting their involvement in defense reactions that could favor the plants response making it more resistant to pathogens. The fact that transcript levels of two genes encoding F3H and a TF TT2 were higher in PpERF24-OX-4 compared to wild type under control conditions suggests that rapid production of phenolic defense compounds could lead to less cellular damage produced by the pathogen. Interestingly, higher expression levels of F3H in control plants and in *B. cinerea*- and *C. gloeosporioides*-infected PpERF24 overexpressing lines compared to wild type were confirmed by RT-qPCR, suggesting that the products of this enzyme could increase defenses during fungal infection. These findings are consistent with the reinforcement of *P. patens* cell walls by the accumulation of phenolic and lignin-like compounds after *B. cinerea* and *C. gloeosporioides* infection (Ponce de León et al., 2012; Reboledo et al., 2015). Moreover, in PpERF24-OX plants the accumulation of phenolic compounds derived from this pathway could lead to the accumulation of compounds with antimicrobial activities, contributing to reduce fungal colonization. Several compounds derived from the phenylpropanoid pathway have been detected in *P. patens*, including caffeic acid, coumaric acid, 4-hydroxybenzoic acid, and cafeoylquinic acid, some of which have antimicrobial activities (Erxleben et al., 2012; Richter et al., 2012). The possibility that PpERF24 overexpressing lines produce higher amounts of some of these molecules needs further investigation.

Several genes involved in cell wall remodeling and integrity maintenance were upregulated in PpERF24-OX-4 compared to wild-type plants under control conditions, which could have an impact on cell wall composition and disease susceptibility. Deposition of heteroxylan near the fungal penetration sites in the epidermal cell wall is believed to enhancxe physical resistance to fungal penetration pegs and hence to improve preinvasion resistance (Chowdhury et al., 2017). Moreover, cell wall polymer cross-linking could increase through association with phenolic compounds, and fungal colonization could be delayed so that other defense strategies can be activated (Huckelhoven, 2005).

Several TFs genes such as *ERFs*, *MYBs*, and *PLATZ* showed higher expression levels in PpERF24-OX-4 compared to wild-type control plants, and transcript levels of these TFs increased in both genotypes after *B. cinerea* inoculation. *ERF138* and *ERF106* are both induced in control PpERF24-OX-4 and PpERF24-OX-2 lines compared to wild type, and *ERF106* is induced during *B. cinerea* infection in both transgenic lines and in PpERF24-OX-4 during *C. gloeosporioides* infection. ERF106 and ERF138 belong to a DREB clade and are induced by different types of abiotic stresses (Reboledo et al., 2022). Other genes that were higher expressed in PpERF24-OX-4 compared to wild type were also DREB members, including *PpERF107* (Pp3c4_2680), *PpERF32* (Pp3c13_4270), *PpERF97* (Pp3c3_6420), and *PpERF118* (Pp3c5_4660). Interestingly, two moss-specific genes, *PpERF36* (Pp3c14_8460) and the homolog of PpERF24, *PpERF132* (Pp3c7_10780), showed higher expression levels in PpERF24-OX-4 compared to wild type under control condition. This suggests the existence of regulatory relationships between different ERFs to fine-tune moss immunity.

*MYBs* Pp3c1_42920 and Pp3c13_5070 were upregulated in both control transgenic lines compared to control wild-type plants, and Pp3c1_42920 also showed higher expression levels in *B. cinerea*-infected overexpressing lines. These results suggest that these two MYBs play a function in moss defenses against pathogens. Interestingly, both MYBs are induced with *B. cinerea* and *C. gloeosporioides*, while no induction was detected with abiotic stresses such as drought, heat, phosphate deficiency, or ABA (Reboledo et al., 2022). The role of MYBs in bryophyte immunity was further highlighted with functional studies of *M. polymorpha* MYB14 showing that this TF contributes to defenses against *Phytophthora palmivora* by the activation of flavonoids biosynthesis (Carella et al., 2019).

Among the upregulated DEGs identified under control conditions, we found genes encoding a peroxidase (Prx) and a respiratory burst oxidase (RBOH). *Prx* Pp3c26_3070 expression increased in PpERF24-OX-4 compared to the wild type under control condition and during *B. cinerea* infection. Like in angiosperms, RBOH and peroxidases are associated to the oxidative burst in *P. patens* after chitin treatment (Lehtonen et al., 2012), and mutants in peroxidase Prx34 are more susceptible to fungal pathogens (Lehtonen et al., 2009). The fact that thioredoxin was also upregulated in *B. cinerea*- and *C. gloeosporioides*-inoculated PpERF24-OX-4 compared to the wild type strengthens the importance of redox regulation during moss–pathogen interactions. Moreover, *QOR*, which encodes a flavoprotein that could protect plant tissues from oxidative stress (Heyno et al., 2013), was upregulated in control and fungal-inoculated tissues of both PpERF24 overexpressing plants. Thus, proper control and balancing of redox status probably favor plant defenses to fungal infection observed in PpERF24 overexpressing plants. Interestingly, *CERK1* involved in chitin sensing was upregulated in PpERF24-OX-4 control compared to wild-type control plants. Moreover, higher expression levels of a gene (Pp3c26_950) encoding a LRR receptor-like kinase in control and *B. cinerea*-inoculated PpERF24-OX-4 plants compared to wild type suggest that this receptor may contribute to trigger defense responses. Consistently, overexpression of *CERK1* and LRR receptor-like kinases activated immunity in angiosperms and increased their resistance to fungal pathogens indicating their importance in fungal recognition and activation of defenses (Chen et al., 2020; Block et al., 2021).

The expression levels of other genes related to defense were higher in PpERF24-OX-4 compared to wild-type control plants, including genes encoding a chitinase, β-1,3 glucanase, LTP, and cupin. Similarly, overexpression of *AtERF1*, *AtERF2*, and *AtERF14* activates expression levels of a chitinase (*ChiB*) and a defensin 1.2 (*PDF1.2*) in *A. thaliana* (Solano et al., 1998; Brown et al., 2003; McGrath et al., 2005; Oñate-Sánchez et al., 2007). LTP Pp3c11_18880 was induced in both PpERF4 overexpressing lines compared to the wild type in control and fungal-inoculated conditions. Like in angiosperms where LTP overexpression increases resistance to fungal pathogens including *B. cinerea* (Ali et al., 2020), *P. patens* LTP could increase defenses through antimicrobial activity or by signaling defense mechanisms. Moreover, a gene encoding an SLC40 was upregulated in PpERF24 overexpressing plants compared to the wild type under control conditions and during infection with *B. cinerea* and *C. gloeosporioides*. SLC genes have been previously shown to be target genes of ERF in angiosperm (Liu and Cheng, 2017), although further investigation is needed to understand their precise role in defense.

The jasmonate pathway was activated in PpERF24-OX-4 compared to control plants, evidenced by increased expression of *AOC*, *OPR*, and the TF MYC. In Arabidopsis, MYC2 and related TFs regulate the jasmonic acid (JA) signaling pathway by activating defense gene expression (Gimenez-Ibanez et al., 2017). MYCs are necessary and sufficient for activating the jasmonate pathway in *M. polymorpha* (Peñuelas et al., 2019). Interestingly, JA is not produced in bryophytes and the ligand for the functionally conserved JA-Ile receptor (COI1) is dinor-12-oxophytodienoic acid (dinor-OPDA) (Monte et al., 2018). *P. patens* has OPDA reductase 1/2 (OPR1/2) orthologs, although orthologs for OPR3 involved in JA synthesis were not found (Han, 2017). This pathway participates in *P. patens* defense against *B. cinerea* (Ponce de León et al., 2012). In angiosperms, ERF ORA59 has been suggested to integrate ethylene and jasmonate signaling and regulate resistance to necrotrophic pathogens, including *B. cinerea* (Pré et al., 2008). In conclusion, our results reveal that *P. patens* has ERFs and DREBs with conserved features in the AP2 domain to other bryophytes and angiosperms, while a group of ERF are moss-specific and have distinctive features in this DNA-binding domain. Moss-specific PpERF24 positively regulates moss immunity against fungal pathogen infection by modulating the expression levels of genes involved in defense responses. Further studies on ERFs present only in mosses will contribute to understand their origin and reveal the molecular mechanisms underlying their action, including DNA-binding elements and interacting proteins and target genes that contribute to moss defense. These studies will give further insights into the generation of diversity in the regulation of defense genes by ERF TFs during stress in different plant lineages.

## Data availability statement

The original contributions presented in this study are publicly available in the NCBI under accession number PRJNA821520.

## Author contributions

GR made the overexpressing lines, performed all the experiments, and analyzed the transcriptomic data. AAg and LV participated in phylogenetic analysis. AAl performed the qPCR analysis for biomass and defense gene expression. GR, AAg, LV, AAl, and IP analyzed and interpreted the data and participated in discussions. IP wrote the manuscript. GR, LV, and AAg helped to write the article. All authors read and approved the final version of the manuscript.

## Footnotes

1
https://phytozome-next.jgi.doe.gov/blast-search

2
https://blast.ncbi.nlm.nih.gov/

3
https://phytozome-next.jgi.doe.gov/

4
https://www.biobam.com/omicsbox/

## Supplementary material

Supplementary Figure 7 Subcellular localization of PpERF24 in agroinfiltrated *N. tabacum* leaves. Confocal microscopy images of *N. tabacum* leaves taken 2 days after agroinfiltration. *N. tabacum* leaves were transformed with the pMDC83 vector (GFP alone, upper panels) or with pMDC83-PpERF24-mGFP6his construct (PpERF24 fused to GFP, lower panels). Visible cell nuclei are marked with a white asterisk in bright field and merged panels. From left to right: GFP, chloroplasts, bright field, and merged images. The scale bars represent 50 μm.

Supplementary Figure 8 Generation of PpERF24 overexpression *P. patens* plants. **(A)** Transcript levels of PpERF24 in untreated (control) and *B. cinerea*-inoculated wild-type (Wt) plants, and untreated PpERF24-OX-1 (OX-1), PpERF24-OX-2 (OX-2), PpERF24-OX-4 (OX-4), and PpERF24-OX-7 (OX-7) plants. **(B)** Expression levels of PpERF24 in the overexpressing lines obtained by RT-qPCR. Asterisks indicate a statistically significant difference between the wild-type and overexpressing PpERF24 plants (Student’s *t*-test, ^****^*P* < 0.0001). **(C)** Phenotype of wild-type, PpERF24-OX-2, and PpERF24-OX-4 moss colonies. The scale bars represent 1 cm except in *P. irregulare*-inoculated tissues and the corresponding controls which is 0.5 cm.

Supplementary Figure 9 Symptom development during pathogen treatment in wild-type and PpERF24 overexpressing plants. Symptom development was evaluated after 2 days of treatment; *B. cinerea* and *C. gloeosporioides* spores, *P.c. carotovorum* elicitors, and water (control). In case of *P. irregulare* treatment, plugs with mycelium or PDA (control) were carefully removed for proper visualization. The scale bars represent 1 cm.

Supplementary Figure 10 Pathogen biomass quantification in wild-type and PpERF24 overexpressing plants. *B. cinerea*, *C. gloeosporioides*, and *P. irregulare* DNA levels at 24 hpi, 72 hpi, and 24 hpi, respectively, were estimated by qPCR analysis. Ratios of pathogen to *P. patens* gDNA are presented. The results and standard deviation of three independent triplicate experiments are shown. Asterisks indicate a statistically significant difference between the wild-type and overexpressing PpERF24 plants (Student’s *t*-test, **p* < 0.05, ^**^*p* < 0.01).

Supplementary Figure 11 Enriched gene ontology (GO) biological process and molecular function terms in PpERF24 overexpressing plants. The top 15 enrichment GO terms obtained by comparing DEGs of *B. cinerea*-treated plants or control plants at 24 hpi in wild-type and PpERF24-OX-4 (OX-4) plants are shown, and significance is expressed as -log10 FDR.
