# Supplementary figures and images for: The moss-specific transcription factor PpERF24 positively modulates immunity against fungal pathogens in Physcomitrium patens
Source: Front Plant Sci. 2022 Sep 15;13:908682. doi: 10.3389/fpls.2022.908682 (PMC9520294; doi:10.3389/fpls.2022.908682)

Supplementary Figure 1. Alignment of the *P. patens* AP2 domains of the DREB clades.

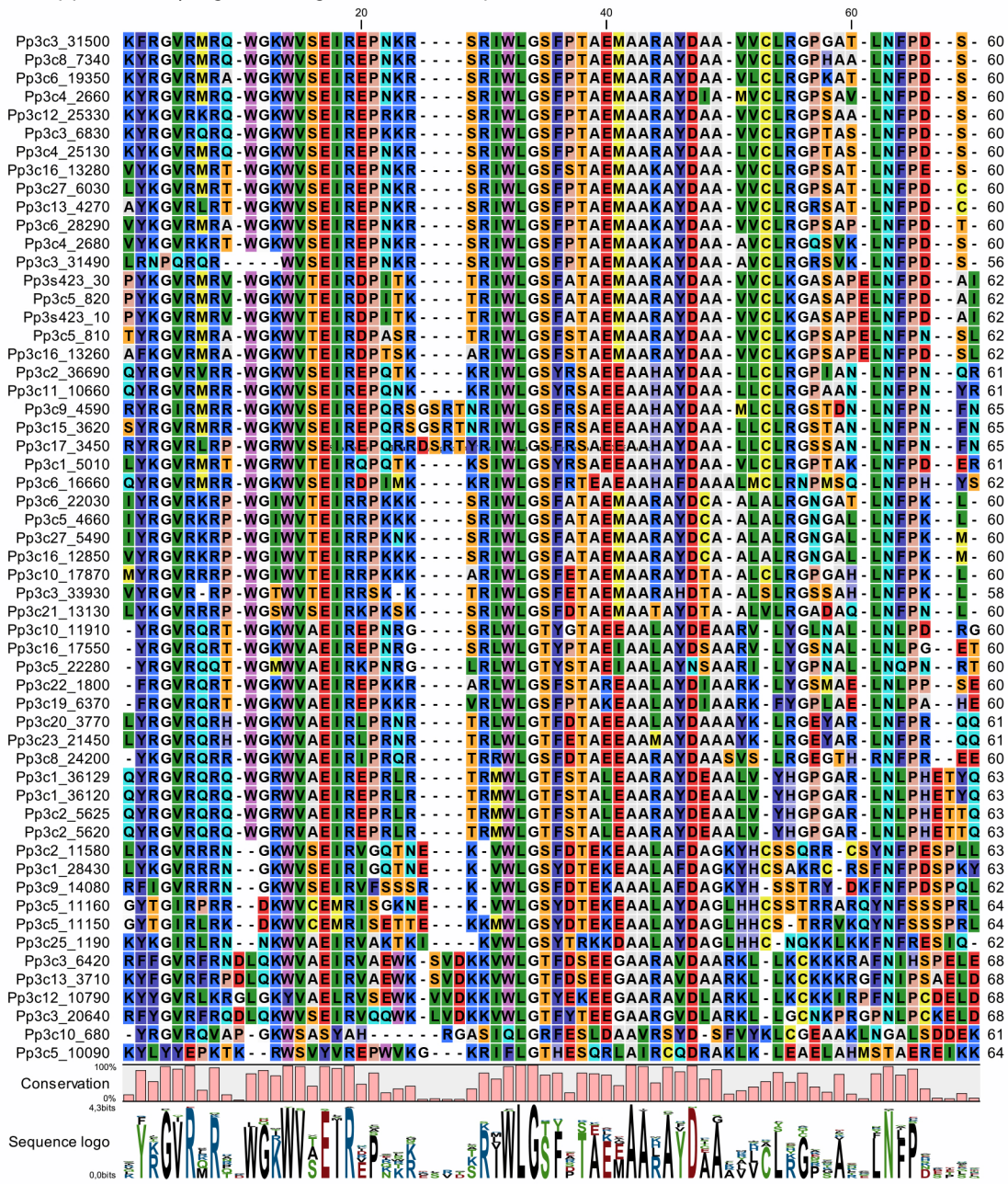

Supplement: Supplementary file 1 [file Image_1.pdf]

Supplementary Figure 2. Alignment of the *P. patens* AP2 domains of the ERF clades.

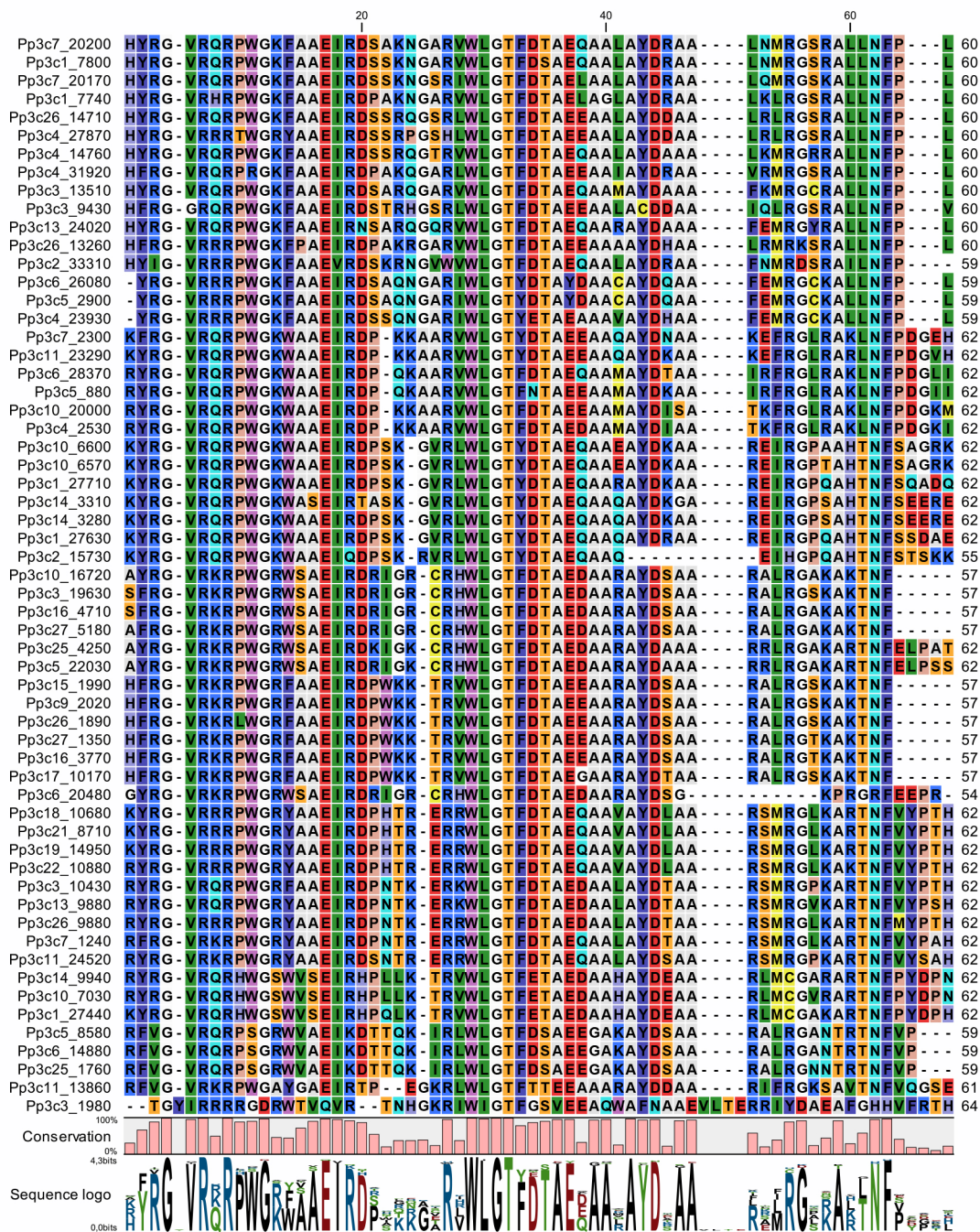

Supplement: Supplementary file 2 [file Image_2.pdf]

Supplementary Figure 3. Alignment of the *A. thaliana* AP2 domain of the DREB clades.

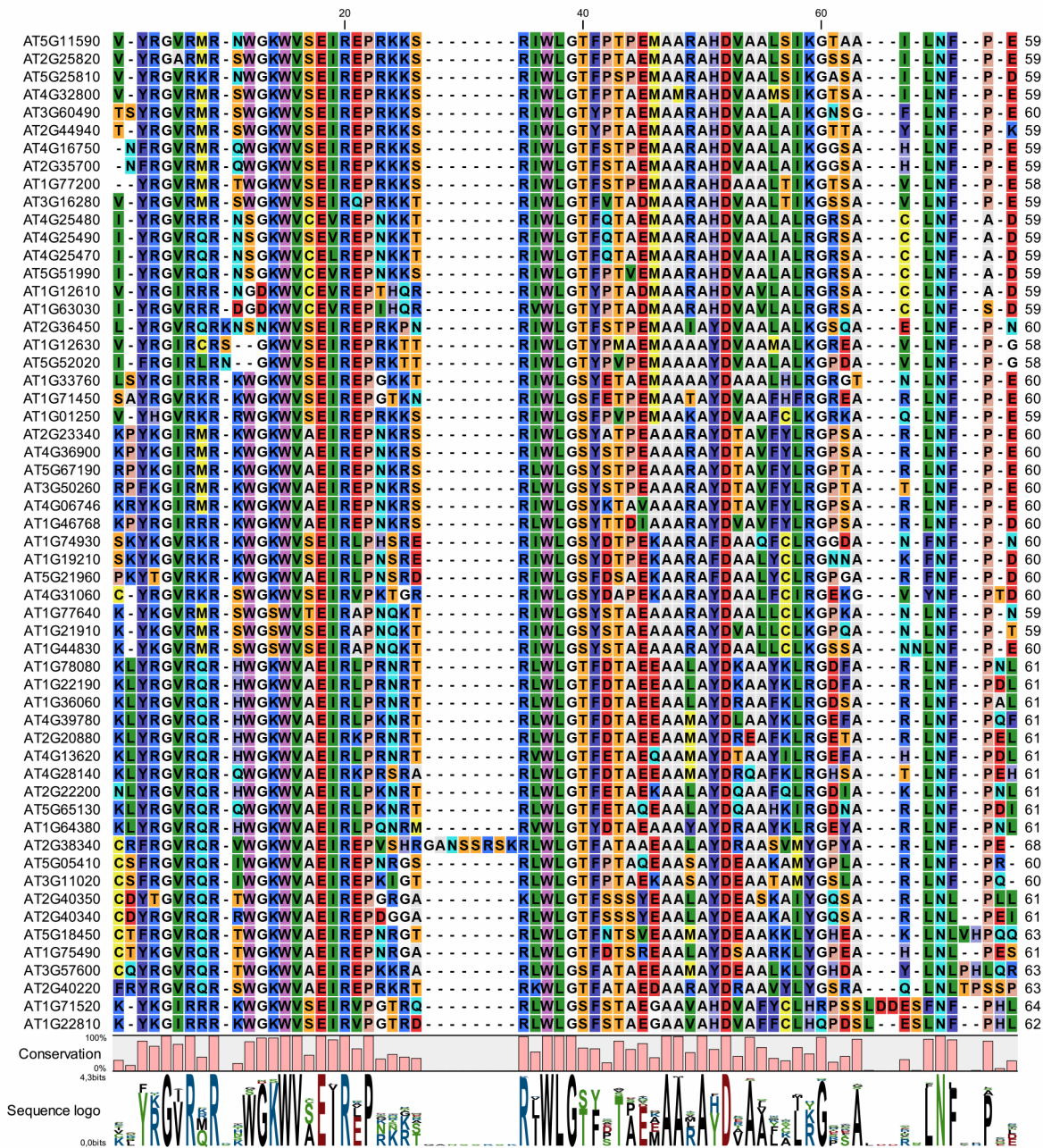

Supplement: Supplementary file 3 [file Image_3.pdf]

Supplementary Figure 4. Alignment of the *A. thaliana* AP2 domains of the ERF clades.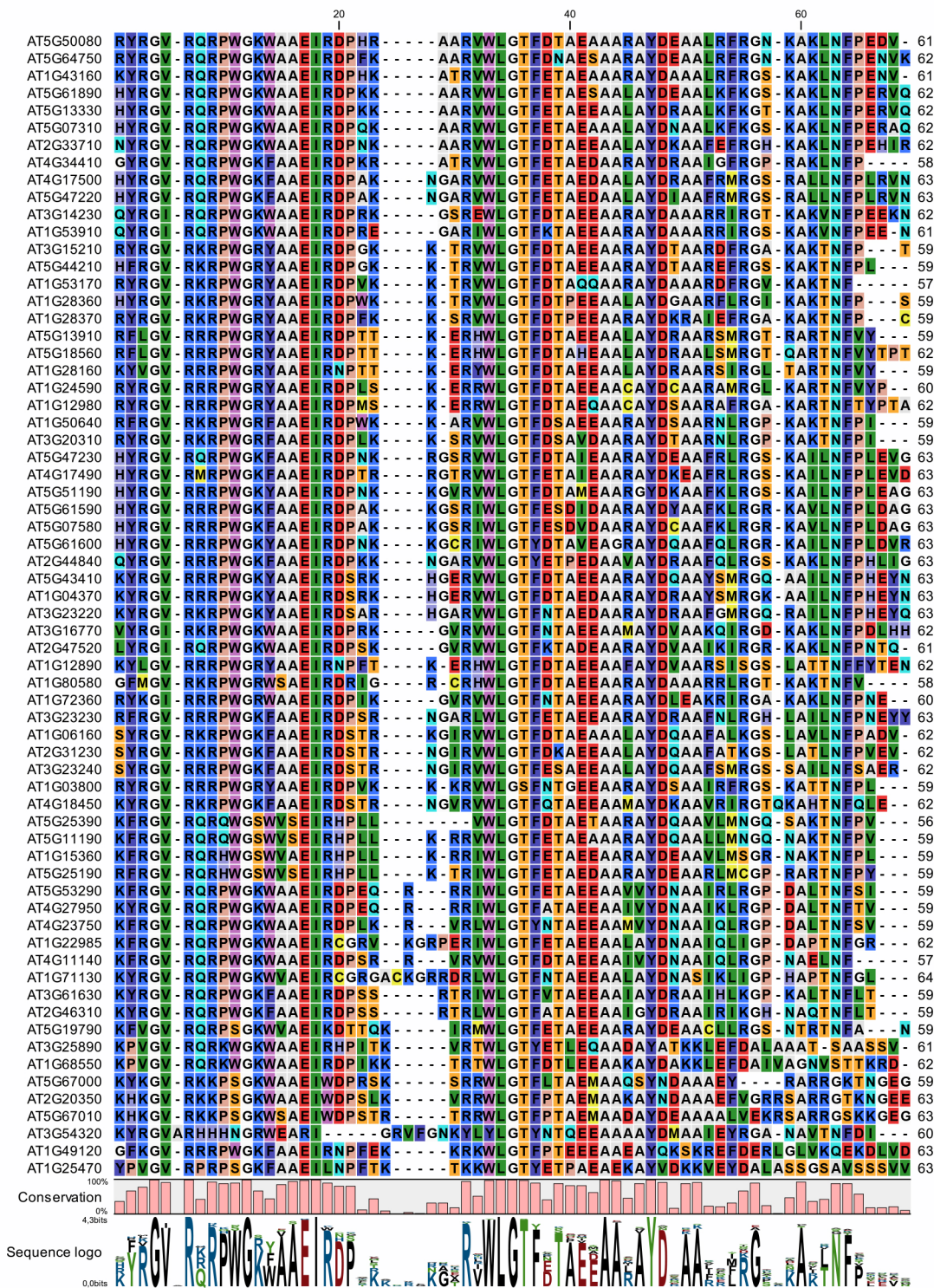

Supplement: Supplementary file 4 [file Image_4.pdf]

Supplementary Figure 5. Alignment of the AP2 domain of the moss-specific clade.

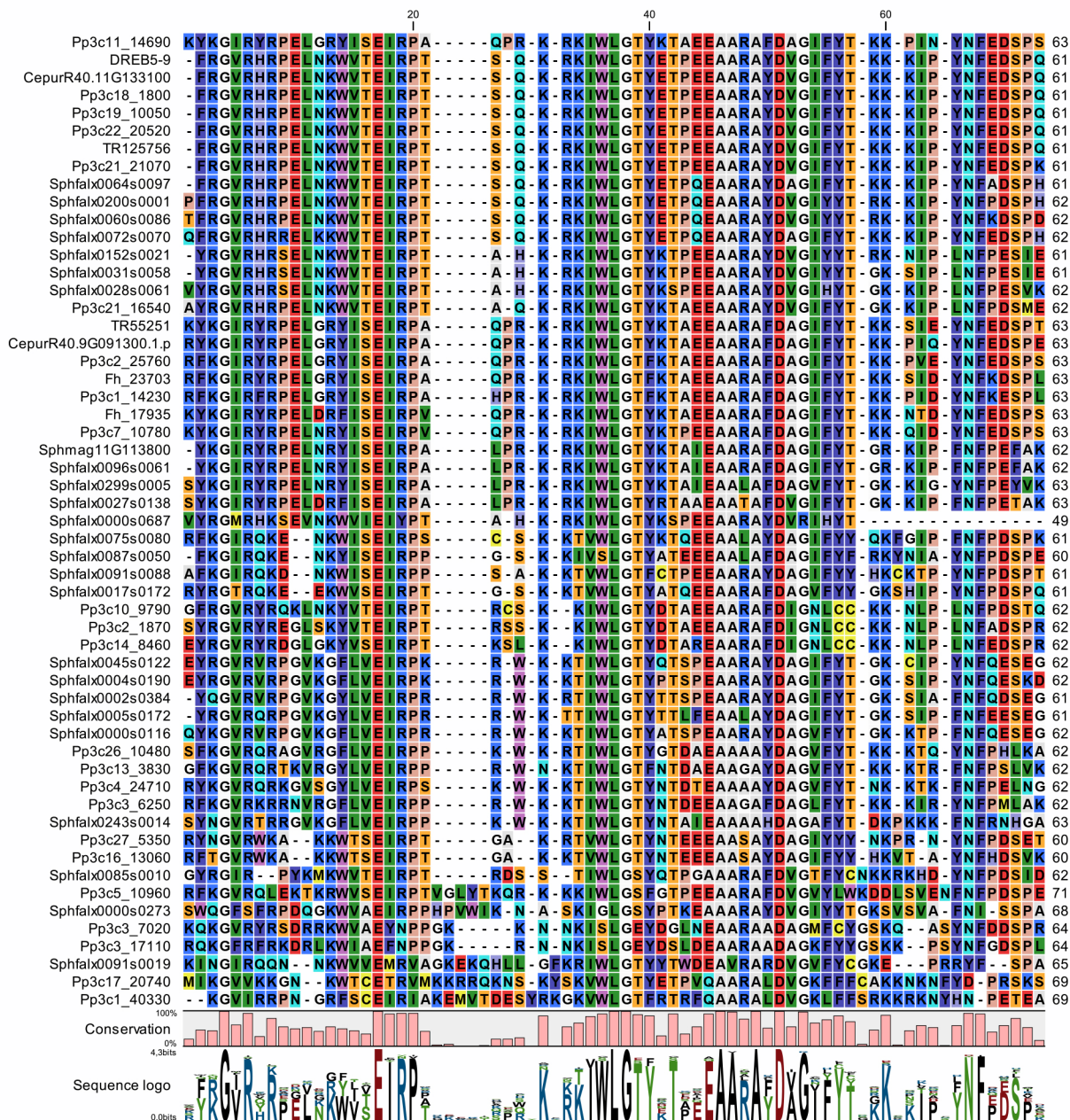

Supplement: Supplementary file 5 [file Image_5.pdf]

Supplementary Figure 6. Alignment of the 12 AP2 domains of moss, hornwort and angiosperms.

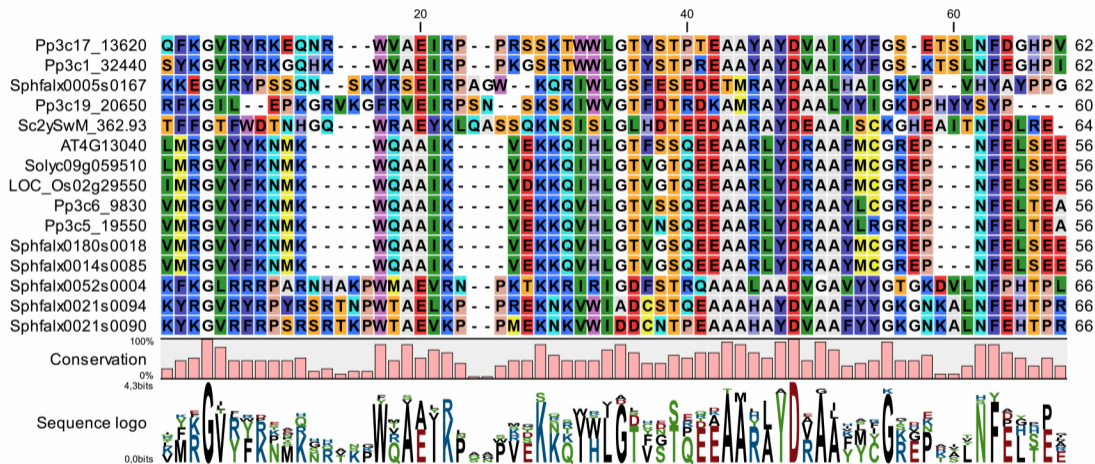

Supplement: Supplementary file 6 [file Image_6.pdf]

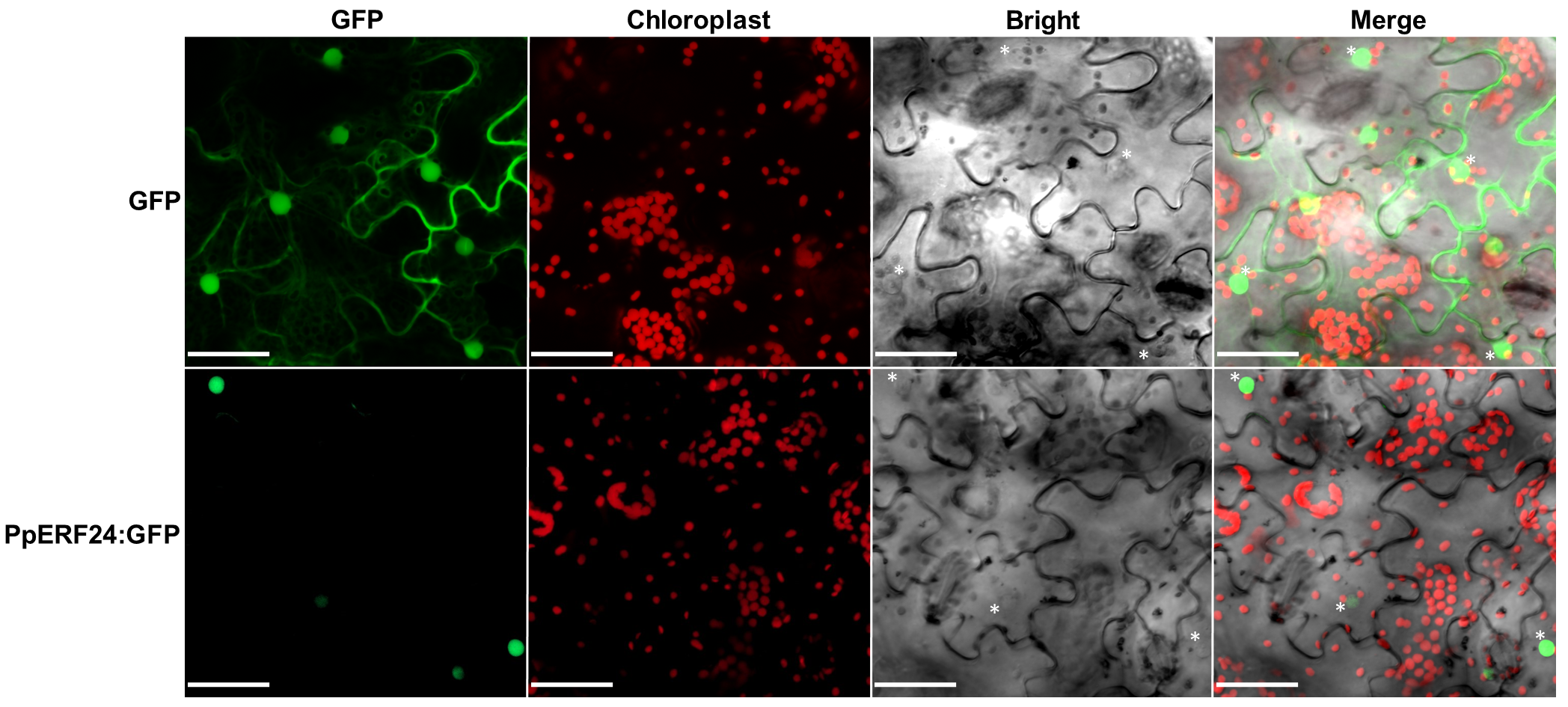

Supplement: Supplementary Figure 7 — Subcellular localization of PpERF24 in agroinfiltrated N. tabacum leaves. Confocal microscopy images of N. tabacum leaves taken 2 days after agroinfiltration. N. tabacum leaves were transformed with the pMDC83 vector (GFP alone, upper panels) or with pMDC83-PpERF24-mGFP6his construct (PpERF24 fused to GFP, lower panels). Visible cell nuclei are marked with a white asterisk in bright field and merged panels. From left to right: GFP, chloroplasts, bright field, and merged images. The scale bars represent 50 μm. [file Image_7.tif]

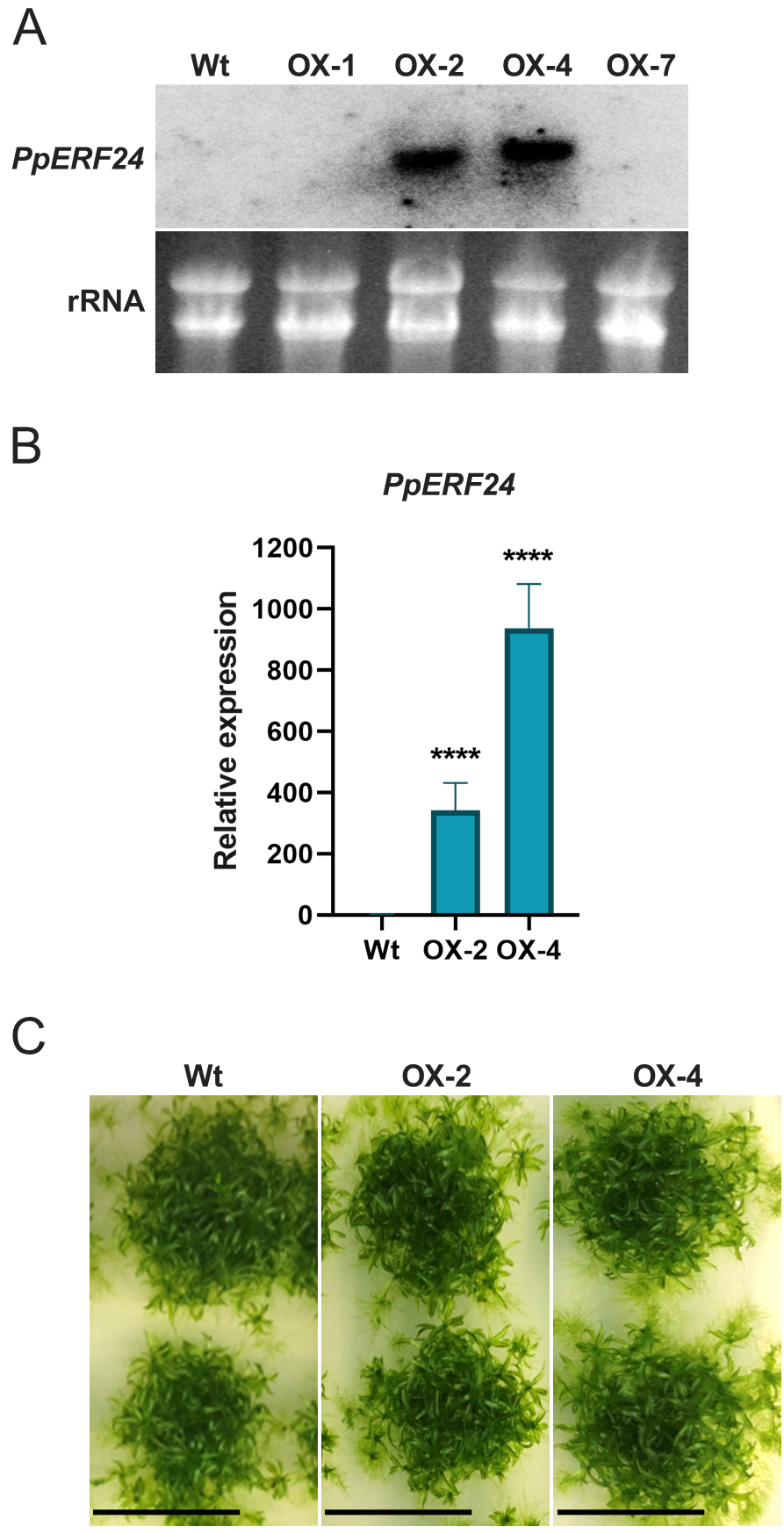

Supplement: Supplementary Figure 8 — Generation of PpERF24 overexpression P. patens plants. (A) Transcript levels of PpERF24 in untreated (control) and B. cinerea-inoculated wild-type (Wt) plants, and untreated PpERF24-OX-1 (OX-1), PpERF24-OX-2 (OX-2), PpERF24-OX-4 (OX-4), and PpERF24-OX-7 (OX-7) plants. (B) Expression levels of PpERF24 in the overexpressing lines obtained by RT-qPCR. Asterisks indicate a statistically significant difference between the wild-type and overexpressing PpERF24 plants (Student’s t-test, ****P < 0.0001). (C) Phenotype of wild-type, PpERF24-OX-2, and PpERF24-OX-4 moss colonies. The scale bars represent 1 cm except in P. irregulare-inoculated tissues and the corresponding controls which is 0.5 cm. [file Image_8.tif]

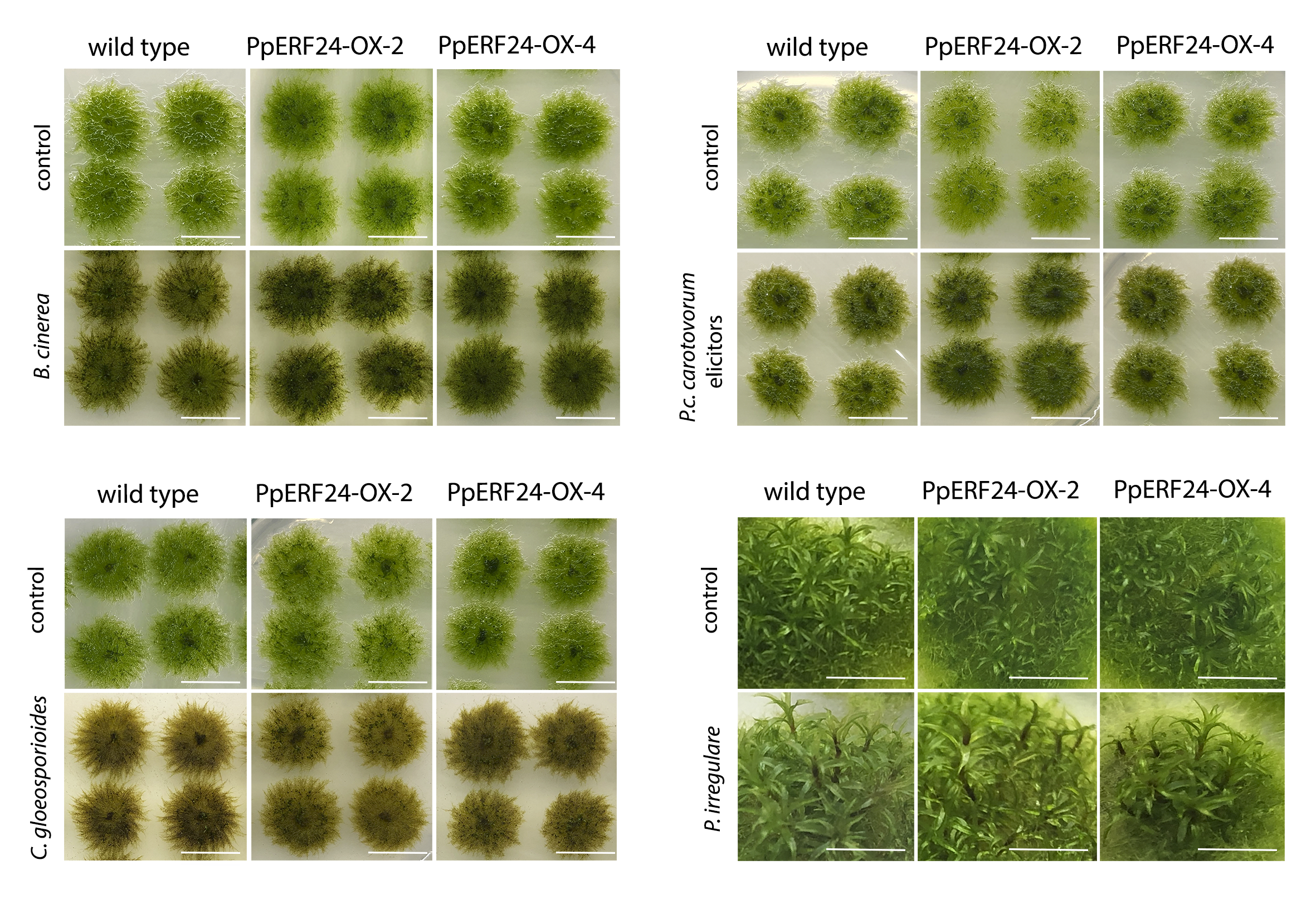

Supplement: Supplementary Figure 9 — Symptom development during pathogen treatment in wild-type and PpERF24 overexpressing plants. Symptom development was evaluated after 2 days of treatment; B. cinerea and C. gloeosporioides spores, P.c. carotovorum elicitors, and water (control). In case of P. irregulare treatment, plugs with mycelium or PDA (control) were carefully removed for proper visualization. The scale bars represent 1 cm. [file Image_9.tif]

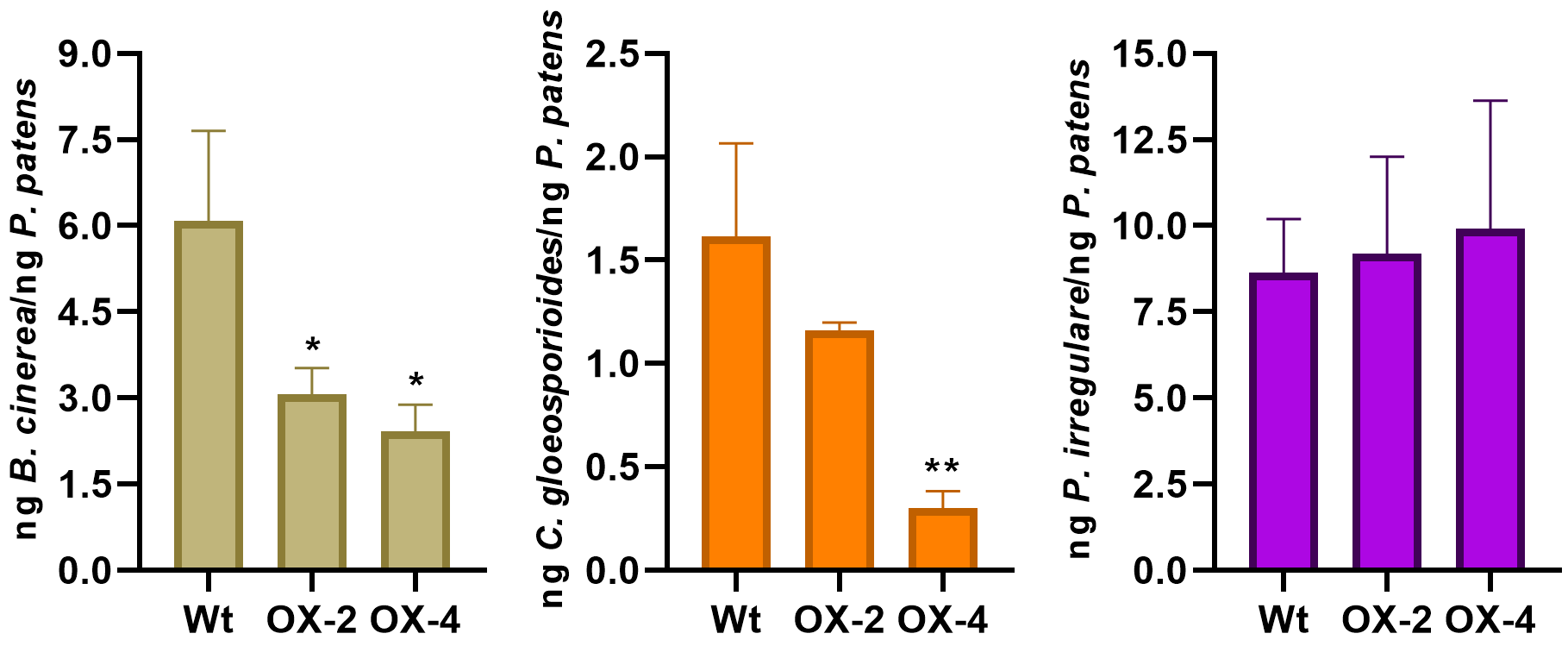

Supplement: Supplementary Figure 10 — Pathogen biomass quantification in wild-type and PpERF24 overexpressing plants. B. cinerea, C. gloeosporioides, and P. irregulare DNA levels at 24 hpi, 72 hpi, and 24 hpi, respectively, were estimated by qPCR analysis. Ratios of pathogen to P. patens gDNA are presented. The results and standard deviation of three independent triplicate experiments are shown. Asterisks indicate a statistically significant difference between the wild-type and overexpressing PpERF24 plants (Student’s t-test, *p < 0.05, **p < 0.01). [file Image_10.tif]

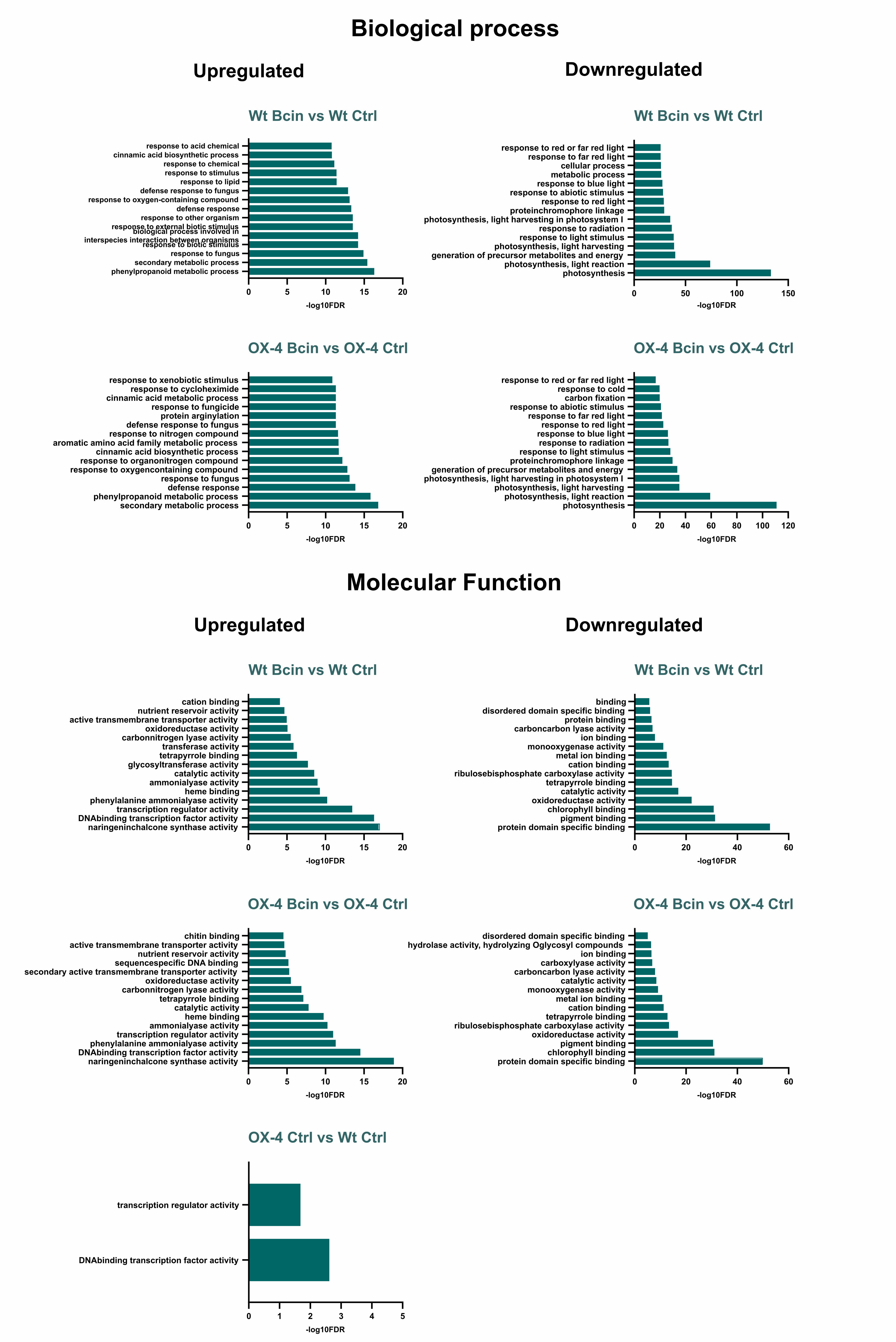

Supplement: Supplementary Figure 11 — Enriched gene ontology (GO) biological process and molecular function terms in PpERF24 overexpressing plants. The top 15 enrichment GO terms obtained by comparing DEGs of B. cinerea-treated plants or control plants at 24 hpi in wild-type and PpERF24-OX-4 (OX-4) plants are shown, and significance is expressed as -log10 FDR. [file Image_11.png]
